# The Formation of Morphologically Stable Lipid Nanocarriers for Glioma Therapy

**DOI:** 10.3390/ijms24043632

**Published:** 2023-02-11

**Authors:** Rais Pavlov, Elvira Romanova, Denis Kuznetsov, Anna Lyubina, Syumbelya Amerhanova, Alexandra Voloshina, Daina Buzyurova, Vasily Babaev, Irina Zueva, Konstantin Petrov, Svetlana Lukashenko, Gulnara Gaynanova, Lucia Zakharova

**Affiliations:** Arbuzov Institute of Organic and Physical Chemistry, FRC Kazan Scientific Center, Russian Academy of Sciences, 420088 Kazan, Russia

**Keywords:** liposome, cerasome, blood–brain barrier, Tween 80, paclitaxel, T98G cells, surfactant, cytotoxicity

## Abstract

Cerasomes are a promising modification of liposomes with covalent siloxane networks on the surface that provide outstanding morphological stability while maintaining all the useful traits of liposomes. Herein, thin film hydration and ethanol sol injection methods were utilized to produce cerasomes of various composition, which were then evaluated for the purpose of drug delivery. The most promising nanoparticles obtained by the thin film method were studied closely using MTT assay, flow cytometry and fluorescence microscopy on T98G glioblastoma cell line and modified with surfactants to achieve stability and the ability to bypass the blood–brain barrier. An antitumor agent, paclitaxel, was loaded into cerasomes, which increased its potency and demonstrated increased ability to induce apoptosis in T98G glioblastoma cell culture. Cerasomes loaded with fluorescent dye rhodamine B demonstrated significantly increased fluorescence in brain slices of Wistar rats compared to free rhodamine B. Thin film hydration with Tween 80 addition was established as a more reliable and versatile method for cerasome preparation. Cerasomes increased the antitumor action of paclitaxel toward T98G cancer cells by a factor of 36 and were able to deliver rhodamine B over the blood–brain barrier in rats.

## 1. Introduction

The problem of increasing the effectiveness of drugs can be solved either by the synthesis of new substances with a certain type of activity or by the design of delivery systems for well-known medicines [1]. Nowadays, a huge fraction of pharmaceutical research revolves around liposomes, owing to their versatility, tunability and vast modification possibilities [2,3,4,5]. Liposomal drug delivery systems have great potential to overcome the problems of low bioavailability of drugs, their premature degradation, their lack of targeting and the high probability of side effects [6]. There are many successful examples in the literature of the development of liposomal formulations for a variety of disease types, including chronic and oncological diseases [7].

Meanwhile, among alternatives to traditional liposomes, based on natural lipids, various novel vesicular carrier types have been introduced in recent decades, such as niosomes [8], transfersomes [1,9,10], bicelles [11,12] and inorganic and hybrid nanoparticles [13]. One of them that is of interest in this particular work—cerasome—was developed and studied in the last two decades [11,14,15,16]. Their building blocks are lipid-mimetic compounds conjugated with triethoxysilyl groups that are capable of self-assembly into vesicular structures and consequent polymerization involving silane hydrolysis and consequent siloxane bond formation [17]. This feature allows for the creation of a morphologically stable, surfactant-resistant nanocarrier that can release cargo in a controlled manner. On their own, cerasome-forming lipids (CFLs) stagger cargo release due to the development of covalent bonds on top of the bilayer [18,19], and also provide reinforcement. In fact, cerasomes are the most morphologically durable bilayer nanocarriers known [20]. They are not only stable during storage, but are also resistant against membrane solubilization by surfactant molecules [17], which makes them a very attractive alternative to the liposomes.

Such a building block for the development of drug carriers has attracted a lot of attention, and various conceptual contributions have been made over the last decade. The synthetic three-part design of the lipid allows for many structural modifications to obtain a functionalized molecule [14,17]. So far, some modifications to the original lipid structures have been proposed and studied by different authors, such as a CFL with cholesterol as a hydrophobic domain [21], branched CFLs with varied tail and headgroup amounts [22], amino acid-based CFLs [19], disulfide-bearing CFLs [23], azide-bearing CFLs [15] and porphyrin-conjugated CFLs [24]. For example, it was shown that the CFLs can be structurally tweaked by varying the number of hydrophobic chains and triethoxysilyl groups on a molecule to release drugs at a slower or quicker rate, which is applicable for both the hydrophilic and hydrophobic content [22]. A redox-responsive CFL containing an S-S bond was also developed to enable siloxane coat detachment in the presence of an oxidizing agent for accelerated drug release [23]. Different CFL mixtures with conventional lipids such as phosphatidylcholines were also successfully prepared and studied in terms of stability and drug release rates, also showing some degree of thermal sensitivity [25,26]. Wang et al. substituted the ethoxysilyl headgroup with triphenylphosphonium and using a mixture of the original N-[N-(3-triethoxysilyl)propylsuccinamoyl]dihexadecylamine (CFL16) and the modified CFL for mitochondria-targeting delivery [25]. Liang et al. conjugated a porphyrin fragment into the cerasomes as a photosensitizer for cancer therapy [24], and designed a cetuximab-loaded nanocarrier with porphyrin for colorectal cancer treatment [27].

Like traditional liposomes, cerasomes can be modified with targeting ligands and stealth polymers for precise action and long circulation times to compose a nanocarrier for anticancer medicine or small interfering RNA [27,28,29,30]. For instance, Li et al. studied how pegylated cationic cerasomes were used as siRNA carriers, and found that such nanocarriers could be effectively used to treat liver diseases [28]. Liang et al. used hybrid liposomal cerasomes as anticancer drug delivery vehicles that can be triggered to release the contents with high-intensity focused ultrasound with help of CO_2_ microbubbles encapsulated into the cerasomes [29]. The same group has also applied cerasomes for simultaneous imaging and doxorubicin release for the treatment of cancer [31]. They further developed a complex nanocarrier to deliver chemotherapeutic drugs and oxygen gas to alleviate multidrug resistance and epithelial–mesenchymal transition, which are typical of hypoxia and cause tumor metastasis [16].

Compared to liposomes, cerasomes have been studied much less as potential drug carriers, and to the best of our knowledge, only one work has been published to date that utilizes cerasomes for drug delivery to the brain. Zhang et al. have created cerasomes doped with Tween 80 and loaded with curcumin, which were then studied as a brain drug delivery system on mice, with the assistance of ultrasound-targeted microbubble destruction for blood–brain barrier (BBB) opening [32]. Traditionally, BBB penetration is considered to be best achieved by drug conjugation with proteins or antibodies that are able to pass the BBB on their own, such as human insulin receptor monoclonal antibody [33]. Alternatively, polysorbate-coated nanoparticles are known to enhance the BBB passage of nanocarriers [32,34,35,36].

**Figure 1:**
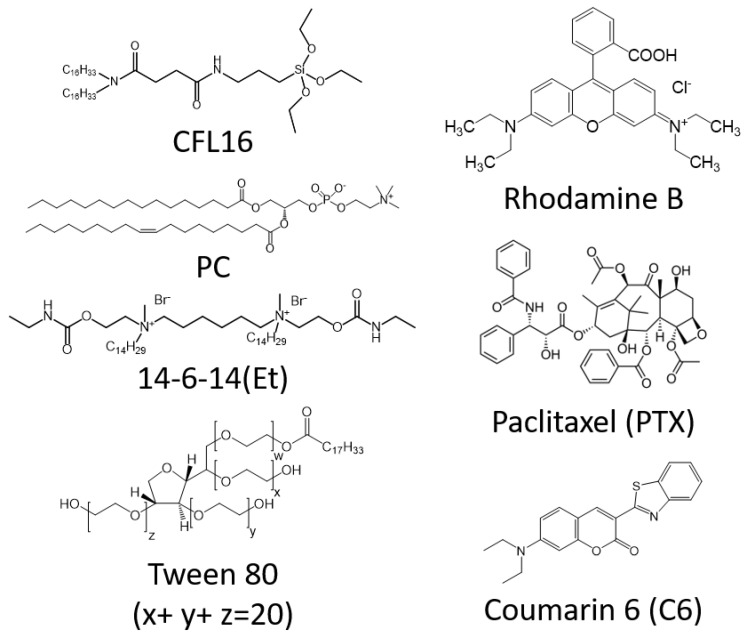
Structural formulas of the compounds used in this work.

The T98G cell line belongs to the family (collection) of a line derived from the glioblastoma of a 61-year-old man [37] that is used as a model for drug development. In this work, for the first time, cerasomes based on CFL16 and phosphatidylcholine (PC) decorated with Tween 80 and cationic gemini surfactant (Figure 1) were formed for the treatment of glioma, a highly aggressive type of cancer that is resistant to chemotherapy [38,39]. For this purpose, two methods for obtaining cerasome were tested (sol injection and thin film hydration). The vehicle with optimal physical and chemical parameters was loaded with paclitaxel (PTX) and was tested for the ability to induce cytotoxicity for T98G glioma cells and to bypass the BBB.

## 2. Results

### 2.1. Cerasomes Obtained via Ethanol Sol Injection Method

By the method of injection and subsequent extrusion, it was possible to obtain aggregates with a hydrodynamic diameter of about 210 nm and PdI in the range of 0.1–0.15 with a zeta potential of −45 mV (Table S1). Since the ability of cerasome-forming lipids to self-organize into vesicles in water depends very much on the degree of hydrolysis of the triethoxysilyl group, the kinetics of hydrolysis of CFL16 in ethanol in the presence of 1 mM HCl was studied by mass spectrometry. It was found that in a solution with a concentration of 1 mg/mL, the lipid undergoes complete hydrolysis in 30 min, and the order of the hydrolysis reaction determined by the van ’t Hoff method is 0.51 (Figure S1). Moreover, for more concentrated solutions (25–100 mM) used to obtain cerasomes, hydrolysis is required for at least 24 h in order to reproducibly obtain good dispersions (Table S1).

**Figure 2:**
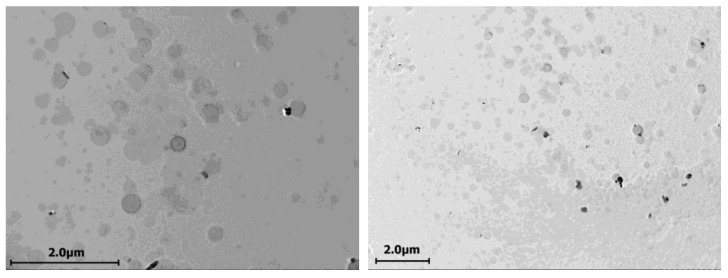
TEM images of CFL16 cerasomes prepared via sol injection method.

Mixed aggregates of the cerasome-forming lipid CFL16 and 1,2-dipalmitoyl-sn-glycero-3-phosphocholine (DPPC) with a molar composition of 1:1 were also obtained by injection. To prepare aggregates with low polydispersity, it is necessary to inject at a temperature higher than the DPPC phase transition temperature (Table S1). TEM images confirmed that cerasomes obtained with the injection method are generally within the sizes of 200–250 nm in diameter and display a clear vesicular structure with hollow lumens (Figure 2).

### 2.2. Cerasomes Obtained via Thin Film Method

The preparation of cerasomes via lipid film hydration method requires the use of a mildly acidic aqueous solution of HCl at pH = 3, which is needed to enable slow hydrolysis of CFL16 molecules for them to become soluble in water for the formation of aggregates. This method yielded unstable results, with some samples obtained according to literature protocols, and some samples that failed to be prepared. Addition of a nonionic surfactant Tween 80 for the induction of BBB-penetrating ability in cerasomes made the preparation protocols more reliable and consistent. Among the prepared cerasomes, which were used further in this study, all of the samples contained an additional 10 mol% of Tween 80 from the total lipid concentration.

Cerasomes formed from CFL16 are usually within 300–400 nm in diameter, with PdI values around 0.3–0.4. Initially, the zeta potential of all samples is positive, due to the fact that particles are formed in a mildly acidic solution with pH = 3. However, upon dilution of the samples with HEPES buffer at pH = 7.4, the zeta potential of all samples shifts to values of −10–−20 mV, governed by the silanol groups’ ionization state at pH = 7.4 (Table 1) [40].

Addition of PC to the CFL16 in the lipid film resulted in the formation of smaller particles with hydrodynamic diameters of 100–250 nm and lower PdI values of 0.2–0.35. As a zwitterionic lipid, PC brings the zeta potential closer to neutral. In general, the addition of PC to the lipid mixture leads to a decrease in hydrodynamic diameter from 333 ± 20 nm for 100% CFL16 cerasomes down to 89 ± 8 nm in case of 100% PC liposomes, while the zeta potential increases from −20 ± 0.3 mV for pure cerasomes up to −7.0 ± 0.5 mV for pure PC liposomes. The addition of 1/35th molar fraction of a cationic surfactant 14-6-14(Et) significantly increases the zeta potential up to 40.7 ± 0.8 mV (Table 1).

**Figure 3:**
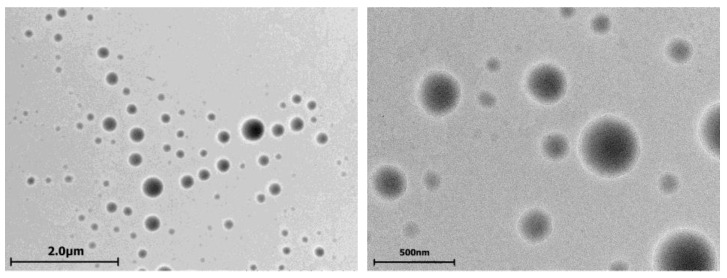
TEM images of 50% CFL16 50% PC cerasomes prepared via thin film hydration method.

TEM analysis of cerasomes formed with the thin film hydration method displays the formation of particles of high sphericity in the range of 20–200 nm in diameter (Figure 3). However, the obtained particles do not resemble vesicular structures and do not have an empty lumen, which means that cerasomes formed with this method are either multilamellar or more closely related to solid lipid nanoparticles.

For the demonstration of cerasome stability, a test using Triton X-100 surfactant was used to dissolve the nanoparticles, with their DLS hydrodynamic diameters and correlogram intercepts monitored initially at 1 h and 24 h of incubation with the 5× excess of surfactant (Table 2). At 1 h of incubation, the observed diameter was not significantly altered (within 50 nm of deviation from the original), and the samples containing 75% and 100% PC as bilayer-forming material demonstrated the most significant increase in size, with the 100% sample failing to produce a meaningful DLS result at 24 h of incubation. The value of the correlogram intercept is a measure of the quality of the size analysis using DLS; optimally, the values should be closer to 1.0, and values above 0.7 usually represent reliable results. In the case of nanoparticles formed with 75% PC and 100% PC, the intercept value is the lowest after 24 h of incubation, which indicates that DLS-distinguishable particles are no longer present in a significant amount.

### 2.3. Evaluation of Cerasome Toxicity

Cerasome cytotoxicity was assessed via MTT assay on T98G glioblastoma cells to highlight their ability to improve the toxic effect of PTX. First of all, unloaded cerasomes of selected compositions with carbamate gemini surfactant as modifiers were tested, and it was found that none of the samples displayed full inhibition at the maximal tested concentration of 1 mM. Generally, cerasome samples not loaded with PTX are two orders of magnitude less toxic than the corresponding samples with PTX. It was also shown that cytotoxicity of all cerasome formulations with PTX is higher than that of free PTX, which means that PTX encapsulation into cerasomes is beneficial for the increased toxicity toward T98G cells (Table 3).

**Figure 4:**
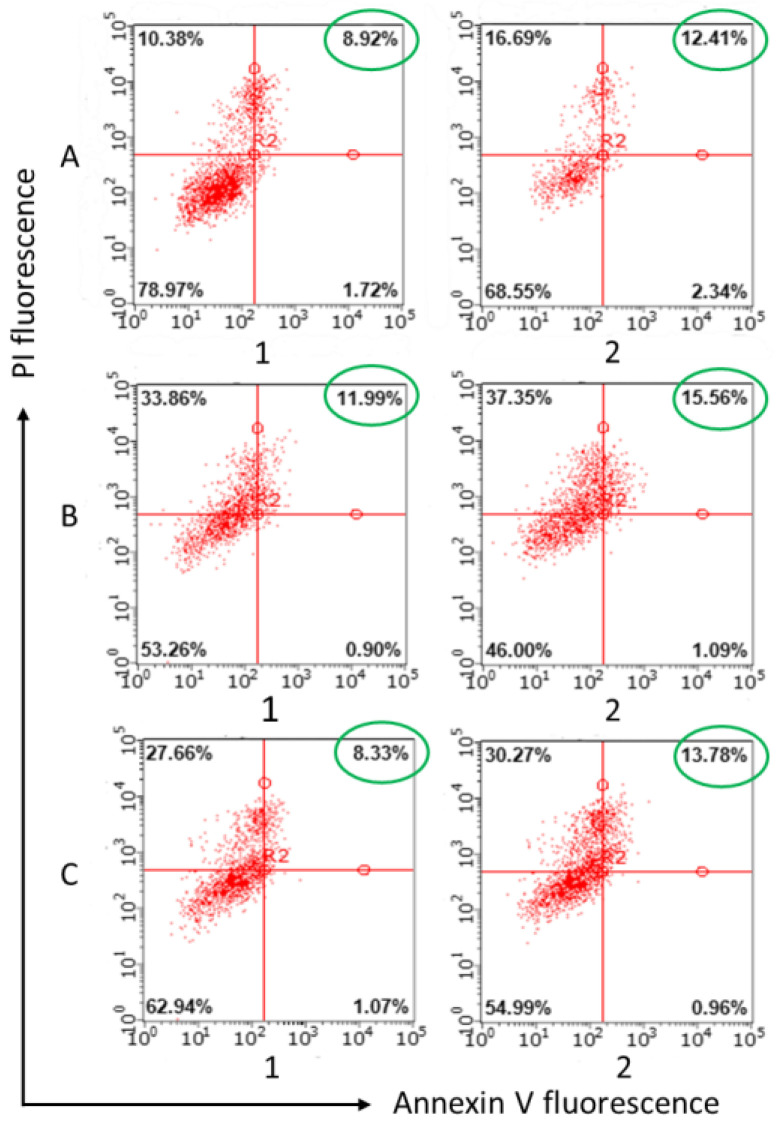
Apoptotic effects measured by flow cytometry using annexin V Alexa Fluor 647 staining protocol. Nanoparticle composition is as follows: (**A**) paclitaxel (PTX); (**B**) CFL 1 mM PC 1 mM PTX 0.08 mM; (**C**) CFL 1 mM PC 1 mM 14-6-14(Et) 0.057 mM and PTX 0.08 mM. Samples were diluted and tested at 0.1 mM of CFL16 (column 1) and 0.2 mM of CFL16 (column 2) or 4 µM and 8 µM for paclitaxel, respectively.

Analysis of apoptosis induction using annexin V Alexa Fluor 647 and propidium iodide fluorescence revealed percentages of apoptotic and dead T98G glioma cells after incubation with unloaded cerasomes, free PTX and PTX cerasomes. Non-PTX-loaded cerasomes induced up to 1–5% of late apoptosis (Figure S2). Clearly, PTX induces apoptotic processes on its own, and the same is observed for cerasome particles loaded with PTX, but at slightly higher rates, increasing from 8.92% to 11.99% at 4 h of incubation for nonsurfactant cerasomes, and from 12.41% up to 15.56% (plain cerasomes) and to 13.78% (cerasomes with surfactant modification) (Figure 4). This confirms that cerasomes are not only able to successfully deliver PTX to the T98G cell interior, but also shows that they increase the proapoptotic action of the anticancer drug.

### 2.4. Cerasome Uptake by T98G Cells

**Figure 5:**
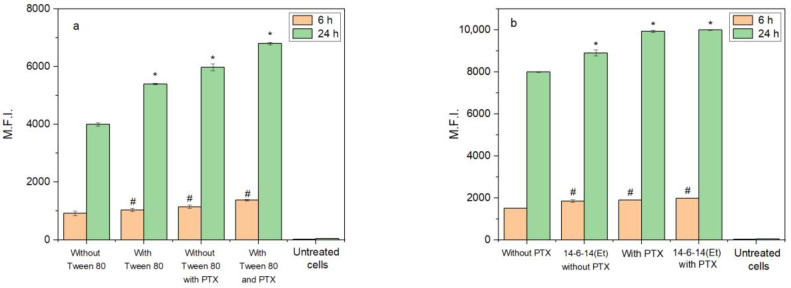
Cellular uptake of cerasomes with and without PTX evaluated using flow cytometry at 6 h and 24 h of incubation: (**a**) comparison of 10% Tween 80 effect on 50% CFL 50% PC cerasomes; (**b**) comparison of 14-6-14(Et) effect on 50% CFL 50% PC cerasomes with additional 10% Tween 80. *—*p* < 0.01 comparing samples at 24 h; #—*p* < 0.01 comparing samples at 6 h. Comparisons were made with first sample in each group.

Flow cytometry was used to evaluate the cellular uptake of cerasomes and the effects of Tween 80 addition, PTX addition and modification with the cationic surfactant 14-6-14(Et) (Figure 5). Cerasomes were doped with coumarin 6 (C6) at the amount of 1/150th from the total lipid concentration as a fluorescent label. Tween 80 addition caused a significant 13% increase of uptake of non-PTX cerasomes at 6 h and 35% at 24 h. PTX addition in this experiment also increased cellular uptake up to an additional 51% and 70% at 6 and 24 h, respectively. For the samples without PTX, the addition of 14-6-14(Et) increases the uptake by about 23% at 6 h, and this difference comes down to only 11% by 24 h of incubation. The samples containing PTX with and without the 14-6-14(Et) surfactant do not significantly differ from each other, but their uptake is higher than for the PTX-free samples. The highest difference of 32% was observed between the 50% PC 50% CFL16 cerasomes without PTX and 50% PC 50% CFL16 + 14-6-14(Et) with PTX at 6 h of incubation. Uptake at 24 h is on average 5.1 times higher than at 6 h.

**Figure 6:**
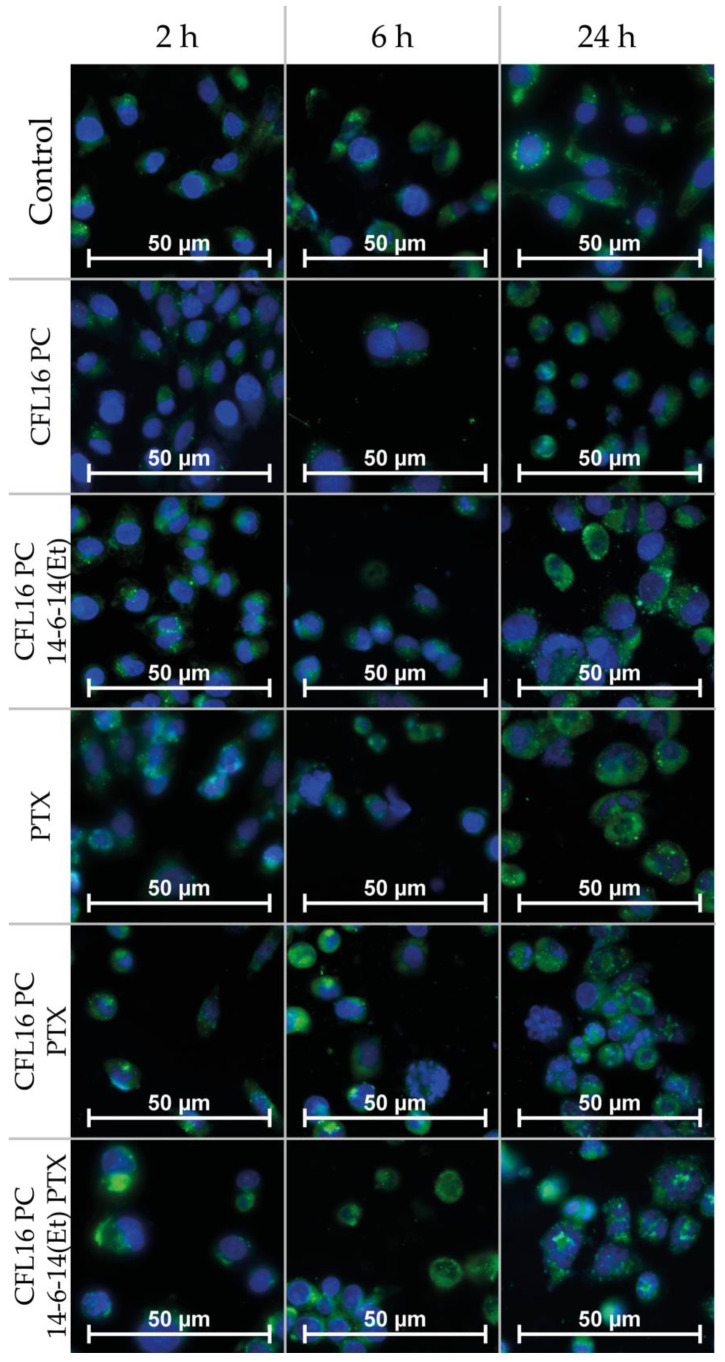
Internalization of CFL PC 1:1 cerasomes into T98G cells at 2, 6 and 24 h after incubation visualized with fluorescence microscopy. Green channel is coumarin 6 and blue channel is DAPI. Separate channel microphotographs are provided in Section S3 of the Supplementary Information.

Fluorescence microscopy was used to study the uptake and apoptotic effects caused by the test systems inside human glioblastoma (T98G) cells after 2, 6 and 24 h of incubation (Figure 6). The nuclei were stained with DNA intercalating dye DAPI (blue fluorescence). C6 (green fluorescence) was used as a fluorescent probe to trace tested systems. Studies were performed at concentrations corresponding to the IC_50_ values of the systems tested. The green fluorescence present in the cytoplasmic regions of cells is indicative of efficient nanoparticle uptake by the cells at all stages of the experiment. Notably, in most cases, localized bubbles of green fluorescence were observed, which may indicate nanoparticle localization in large endosomal and lysosomal compartments. After 2 h incubation of the studied objects, apoptotic effects characteristic of the early stage of apoptosis were observed for the PTX +C6 and CFL16 PC PTX +C6 systems. In the early stages of apoptosis, cells shrivel, losing up to 1/3 of their volume. However, the cells are still able to actively divide. As the incubation time increased to 6 h with the PTX +C6, CFL16 PC PTX +C6 and CFL16 PC +14-6-14(Et) +PTX +C6 systems, apoptotic effects increased. Fragmentation of the nuclei and condensation of the cytoplasm were observed. After 24 h incubation for the PTX +C6, CFL16 PC PTX +C6 and CFL16 PC +14-6-14(Et) +PTX +C6 systems, processes characterizing the stage of late apoptosis were observed. In T98G cells, nuclear fragmentation became clearly visible, the cytoplasm condensed (shriveled), and a significant number of apoptotic bodies were formed. This is generally true for all samples containing PTX after 24 h of incubation (three bottom frames in the third row of Figure 6).

### 2.5. Analysis of Cerasome Compatibility with Blood

Hemolysis tests provide useful information about the compatibility of nanoparticles with blood. In our case, all samples were tested at total concentrations of 0.5 mM and less for nanoparticles and up to 0.1 mM for unencapsulated PTX. All the samples showed less than 50% hemolysis at concentrations of 0.5 mM; the most hemotoxic was cerasome sample with incapsulated PTX without the 14-6-14(Et) cationic surfactant, showing 12.5% of hemolysis at 0.5 mM. Considerably less hemolysis was observed for the cationic cerasome composition with the 14-6-14(Et) surfactant, e.g., 7.7% at 0.5 mM total concentration. An amount of 0.1 mM of PTX induced 11.5% hemolysis (Table S2).

Hemagglutination tests are another way to probe sample compatibility with blood. Even the highest tested concentration, being 0.5 mM for cerasome samples and 0.1 mM for unencapsulated PTX, did not induce agglutination (Figure S3).

### 2.6. In Vivo Assessment of Cerasome Ability to Cross the BBB

**Figure 7:**
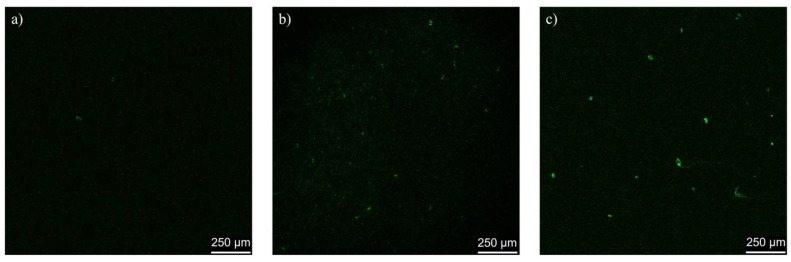
Rat brain slices after administration of (**a**) free rhodamine B; (**b**) plain rhodamine B-loaded cerasomes; (**c**) Tween 80 cerasomes labeled with rhodamine B.

Administration of cerasomes labeled with a fluorescent dye, rhodamine B, demonstrated cerasomes’ ability to penetrate BBB in rats. The images of rat brain slice confocal microscopy reveal very little green fluorescence of rhodamine in the case of free rhodamine B injection and show noticeable green spots, indicating the penetration of rhodamine B-labeled CFL16 PC 1:1 plain cerasomes and the same cerasomes decorated with Tween 80 (Figure 7).

## 3. Discussion

It has been previously shown in the literature that particles formed from CFL16 by the injection method have low polydispersity values in the range of 0.05–0.13 [41], and it is necessary to ensure a sufficiently long incubation time of the cerasome-forming lipid in acidified ethanol, at least 12 h. However, as reported in the literature, the particles obtained by this method were also visualized using electron microscopy, and fusion of particles into large clusters was observed as it was in this work, apparently due to the covalent crosslinking of silanol groups on the surface of the particles [14].

By the method of thin film hydration, particles obtained were similar to those in [29], where the authors used the same method to obtain cerasomes containing a significant proportion of phospholipids in their composition; the mean diameters of particles were evaluated to be ranging from 170–190 nm. Additional DLS analysis of different hybrid cerasome samples also showed that the increase in PC fraction in the lipid mixture leads to the decrease in diameter (Table 1). Quantitative analysis of images from transmission electron microscopy showed the presence of a large number of particles with diameters smaller and bigger than the diameter of 150 nm observed by dynamic light scattering. According to analysis of TEM data (distribution is shown on Figure S4), a significant number of particles of 20–100 nm and 200–400 nm in diameter were also present, which is in accordance with the polydispersity index of around 0.25.

Cerasome dissolution stability and the ability to resist solubilization with Triton X-100 has shown that the 50% PC 50% CFL16 particles are optimal, while resulting in the smallest diameters, which are beneficial for cellular uptake, and still showing stability against Triton X-100 solubilization. It is safe to say that high stability against surfactant dissolution was observed in the case of CFL-rich samples, containing 60%, 70, 80% CFL16; however, each of these samples contained particles larger than 150 nm in diameter, which is not optimal for the purposes of drug delivery [42]. This meant that the mixture of 50% CFL16 and 50% PC, which was the smallest, while also being resistant to surfactant dissolution, was optimal for upcoming in vitro and in vivo experiments.

Interestingly, in an attempt to load the injection-type cerasomes with PTX, the drug was dissolved in the ethanol solution with CFL16 prior to injection; however, upon preparation, a significant amount of precipitate was always observed, indicating that PTX loading was subpar. On the other hand, cerasomes prepared by the method of thin film hydration were loaded with PTX with no apparent precipitates formed, which indicated complete loading of the drug.

The cytotoxicity of non-PTX cerasomes both of anionic and cationic (with surfactant) nature was considerably lower than for PTX-loaded cerasomes, with IC_50_ values hovering around 0.5 mM of total lipid concentration (PC + CFL16). Importantly, PTX encapsulation in cerasomes dramatically increased the cytotoxicity of cerasomes, lowering their IC_50_ values down to 4–5 µM. While free PTX IC_50_ values were registered at 8.8 µM, the PTX content of 1/25th of total lipid molar quantity in cerasomes meant that the active concentration of the chemotherapy agent was around 160–200 nM when encapsulated into the nanoparticles. This is around 36 times more cytotoxic than free PTX, which shows the significance of its encapsulation in cerasomes. Cell apoptosis assay showed slightly higher apoptosis rates for PTX-loaded cerasomes than for free PTX as well. A slightly more intensive cellular uptake was also observed for PTX-loaded cerasomes compared to cerasomes without PTX, which may be explained by increased cellular membrane permeability induced by the toxicity of PTX. The cationic gemini surfactant 14-6-14(Et) has been previously used to enhance cellular uptake of liposomes by PC-3 cancer cells, where a correlation between nanoparticle zeta potential and their uptake was observed [43]. In our case, however, the cationic charge did not significantly affect cytotoxicity or cellular uptake. The addition of Tween 80, however, did result in a significant increase in cellular uptake of 35% after 24 h of incubation, which may be attributed to the fact that Tween 80 cerasomes are more morphologically stable and less prone to aggregate.

Fluorescence microscopy analysis was conducted at fixed periods of incubation at 2, 6 and 24 h. The results firstly confirm the internalization of cerasomes labeled with Coumarin-6 green fluorescence; they can be detected in abundance in the cytoplasmic regions of the cells, with a significant amount localized around the cellular membranes. A significant portion of green fluorescence is observed in small spherical clusters that are most likely lysosomes. In the PTX-containing samples, apoptotic processes are evident as nuclei are disrupted into small bodies. For the CFL16 PC +C6 system, a slight apoptosis-inducing effect appeared only after 24 h. For the CFL16 PC +14-6-14(Et) + C6 system, processes more resembling necrosis were observed (the nuclei increased in size and collapsed). These results, along with cytotoxicity tests, show that PTX action is greatly enhanced by the use of nanocarriers.

The BBB penetration assay was conducted to confirm cerasomes’ ability to enter brain tissue with Tween 80 modification. It is known that Tween 80 headgroups attract apolipoprotein E in the bloodstream, which is then responsible for BBB penetration [35]. This approach was also successfully used in the development of the treatment for Parkinson’s disease with cerasomes previously [32], and is also in line with the current state of the art of nanomedicine targeting, which takes into account the in vivo conditions of opsonization for robust delivery assessment [44]. However, in our case, Tween-free cerasomes also caused green fluorescence in rat brain (Figure 7), which means that the probe encapsulation plays a more important role than Tween 80.

Both rhodamine and paclitaxel are subject to efflux from BBB endothelial cells due to P-glycoprotein (P-gp) action, which is a membrane protein in the luminal side of endothelial cells that helps them filter out certain molecules and prevent them from crossing the BBB [45]. It is for this reason that P-gp inhibitors enhance the cytotoxic effect and antitumor action of paclitaxel in the brain. In case of cerasomal delivery, no inhibition of P-gp is expected, and rhodamine B is protected from efflux because it is loaded inside the cerasome particles, which facilitate its transport through endothelial cells to the brain tissue. Rhodamine B, which was used in this work, is a charged molecule, which makes it difficult for it to pass through the BBB on its own [46], especially compared to its more hydrophobic counterpart Rhodamine 123, which is a known P-gp substrate [47]. This is evidenced by the absence of luminescence on brain sections after the injection of free rhodamine into mice.

To sum up, cerasomes as a drug delivery vehicle were evaluated for their potential to treat glioma. Firstly, cerasomes prepared via two different methods—injection and thin film hydration—were compared based on their size and morphology, and thin film cerasomes were more promising since they were more uniform, as evident by TEM, and able to encapsulate PTX without precipitation. Secondly, the utilization of hybrid 50% CFL16 and 50% PC cerasomes was able to improve PTX cytotoxicity toward the T98G glioblastoma cell line by a factor of 36, making it a very effective nanomedical tool to treat glioma. Finally, the BBB penetration ability of cerasomes labeled with rhodamine B was confirmed in rats using confocal microscopy of rat brain tissue. Overall, cerasomes were able to be successfully modified for the purpose of delivery of PTX over the BBB as conventional liposomal nanocarriers, and also possessed an invaluable characteristic of utmost morphological stability, which makes them a promising candidate for the treatment of glioma and the development of novel nanomedical formulations.

## 4. Materials and Methods

### 4.1. Materials

Soybean phosphatidylcholine (PC) was a gift from Lipoid GmbH (Ludwigschafen, Germany) and 1,2-dipalmitoyl-sn-glycero-3-phosphocholine (DPPC), rhodamine B, 1-Hydroxybenzotriazole hydrate (HOBt), N-(3-Dimethylaminopropyl)-N′-ethylcarbodiimide hydrochloride (EDC), triethylamine (Et_3_N), succinic anhydride, 1-bromohexadecane, Rhodamine B, 4-(2-Hydroxyethyl)piperazine-1-ethanesulfonic acid (HEPES), Coumarin 6, 3-(4,5-dimethylthiazol-2-yl)-2,5-diphenyltetrazolium bromide (MTT), 4′,6-diamidino-2-phenylindole (DAPI), paraformaldehyde powder were purchased from Sigma-Aldrich (St. Louis, MO, USA). Eagle nutrient medium from Chumakov Institute of Poliomyelitis and Virus Encephalitis, nonessential amino acids DMSO and fetal calf serum were from PanEco (Moscow, Russia). Tween 80 was from Ferak Berlin GmbH (Berlin, Germany). Abovementioned chemicals were used without additional purification. Chloroform was from EKOS (Moscow, Russia) and was dried over CaCl_2_ for 48 h and distilled before use. Cerasome-forming lipid N-[N-(3-triethoxysilyl)propylsuccinamoyl]dihexadecylamine (CFL16) was synthesized by a 2-step condensation of dihexadecylamine with succinic anhydride with Et_3_N and (3-aminopropyl)triethoxysilane with HOBt and EDC in dichloromethane according to [23]. The obtained compound was characterized using AmazonX ion trap mass spectrometer (Bruker Daltonic GmbH, Bremen, Germany) (Figures S6, S8 and S10) and Bruker Avance 400 NMR-spectrometer (Bruker Daltonic GmbH, Bremen, Germany) (Figures S5, S7 and S9).

### 4.2. Cerasome Preparation

In the injection method, stock solutions of CFL16 were prepared in acidified ethanol as described in [41]. The solutions were first incubated for at least 12 h at room temperature and then slowly injected into Milli-Q water with intensive stirring using an automatic pipette; the vessel was thermostatted in a water bath. The samples were stored overnight before further analysis. In cases of mixed compositions of CFL16 with DPPC, the lipid was dissolved in the acidified ethanol as well.

In thin film method, stock solutions of film components in chloroform were prepared in the following concentrations: CFL 20 mM, PC 45 mM, Tween 80 5 mM, PTX 1 mM, C6 1 mM, 14-6-14(Et) 3 mM. To form lipid films, all stock solutions in quantities calculated for 3 mL of the final aqueous solution were added to empty vessels. Chloroform was entirely removed from the mixture by vacuum rotary evaporation (RE-52AA rotary evaporator, Shanghai Jingke Scientific Instrument Co., Ltd., Shanghai, China). Then, the dried lipid films were hydrated with 2 mL 1 mM HCl or solution of 0.5 mg/mL rhodamine B in 1 mM HCl. Cerasomes were prepared by vortex mixing (ZX3 Advanced Vortex Mixer, VELP Scientifica, Usmate Velate, Italy) 5 times over the span of 2 h for the duration of 1 min each time, then the particles were ultrasonically dispersed by Bandelin Sonopuls HD 4100 device (Berlin, Germany) with a power of 10 W for 8.5 min. The resulting 2 mL solutions were diluted with 50 mM HEPES buffer pH = 7.4 up to 3 mL and stored at 4 °C.

### 4.3. Cerasome Characterization

The particle size, polydispersity index and zeta potential were determined by dynamic and electrophoretic light scattering on Anton Paar LiteSizer 500 (Graz, Austria) device and Calliope software. The data were obtained in a series of 3 measurements; the measurement angle was 175 degrees and undiluted samples were used. Correlograms obtained with DLS are provided in the Section S2 of the Supplementary Materials.

Transmission electron microscopy (TEM) images were obtained at the interdisciplinary center “Analytical microscopy” of Kazan Federal University, using a Hitachi HT7700 Exalens microscope (Tokyo, Japan). The images were acquired at an accelerating voltage of 100 kV. Samples were dispersed on 300 mesh 3 mm copper grids (Ted Pella, Redding, CA, USA) with continuous carbon-formvar support films.

### 4.4. Cell Culture

For the experiments, we used cancer cell cultures of glioblastoma cell line (T98G) from the collection of the Institute of Cytology, Russian Academy of Sciences (St. Petersburg, Russia). The cells were cultured in a standard Eagle’s nutrient medium and supplemented with 10% fetal calf serum and 1% nonessential amino acids. The cells were seeded into a 96-well plate (Nunc) at a concentration of 1 × 10^5^ cells/mL, 150 μL of medium per well, and cultured in a CO_2_ incubator at 37 °C. Twenty-four hours after seeding the cells into wells, the compound under study was added at a preset dilution, 150 μL to each well. The dilutions of the compounds were prepared immediately in nutrient media; the 5% DMSO that did not induce inhibition of cells at this concentration was added for better solubility.

### 4.5. Cytotoxicity Assessment via MTT-Assay

The cytotoxic effect on cells was determined using the MTT test. Cells were seeded on a 96-well Nunc plate at a concentration of 5 × 10^3^ cells per well in a volume of 100 µL of medium and cultured in a CO_2_ incubator at 37 °C until a monolayer was formed. Then, the nutrient medium was removed and 100 µL of solutions of the tested composition in the given dilutions were added to the wells, which were prepared directly in the nutrient medium with the addition of 5% DMSO to improve solubility. After 24 h of incubation of the cells with the test compounds, the nutrient medium was removed from the plates and 100 µL of the nutrient medium without serum with MTT at a concentration of 0.5 mg/mL was added and incubated in a CO_2_ incubator for 4 h at 37 °C. After incubation, the medium with MTT was removed and 100 μL of DMSO was added to each well to dissolve the formed formazan crystals. Optical density was recorded at 540 nm on an Invitrologic microplate reader (Novosibirsk, Russia). The experiments for all compounds were repeated three times. Calculation of IC_50_, the concentration of the test compound that causes suppression of cell growth by 50%, was made using the program: MLA—“Quest Graph™ IC_50_ Calculator”. AAT Bioquest, Inc. (Pleasanton, CA, USA).

### 4.6. Induction of Apoptotic Effects

T98G cells at 1 × 10^6^ cells/well in a volume of 2 mL were seeded into 6-well plates. After 24 h of incubation, various concentrations of test compounds were added to wells. The cells were harvested at 2000 rpm for 5 min and then washed twice with ice-cold PBS, followed by resuspension in binding buffer. Next, the samples were incubated with 5 μL of annexin V Alexa Fluor 647 (Sigma-Aldrich, St. Louis, MO, USA) and 5 μL of propidium iodide for 15 min at room temperature in the dark. Finally, the cells were analyzed by flow cytometry (Guava easy Cyte, Merck, Rahway, NJ, USA) within 1 h. The experiments were repeated three times.

### 4.7. Flow Cytometry Measurement of Cell Association

T98G cells at 1 × 10^6^ cells/well in a volume of 2 mL were added to 6-well plates and cultured in a CO_2_ incubator at 37 °C in an atmosphere containing 5% CO_2_ until a monolayer was formed. Then, the nutrient medium was taken and solutions in the nutrient medium of various concentrations of the studied compositions were added to the wells. The plates were cultured in a CO_2_ incubator at 37 °C in an atmosphere containing 5% CO_2_ for 24 h. Coumarin 6 dye was used as a fluorescent probe. Treated cells were analyzed by flow cytometry (Guava easy Cyte, Merck, Rahway, NJ, USA). The experiments were repeated three times and data were reported as mean fluorescence intensity (M.F.I.) values ± standard deviation.

### 4.8. Fluorescence Microscopy

T98G cells at 1 × 10^5^ cells/well were plated in 6-well plates with coverslips at the bottom of each well. After 24 h of incubation, test systems were introduced into the wells at a concentration of 1.5 µM of CFL16 and cultivated for 2, 6 and 24 h in a CO_2_ incubator. Then, after treatment with test systems, T98G cell samples were dried, fixed with 4% paraformaldehyde for 30 min, and stained with 0.001% DAPI with an excitation peak at 359 nm and an emission peak at 457 nm for 1 h, washed with PBS buffer pH 7.4. The studies were carried out on a luminescent microscope Nikon Eclipse Ci-S (Nikon, Tokyo, Japan) at a magnification of 400×.

### 4.9. In Vivo Analysis of BBB Penetration Using Confocal Microscopy of Brain Slises

All experiments with animals were carried out in accordance with the Directive of the Council of the European Union 2010/63/EU. The experimental protocols were approved by the Animal Care and Use Committee of the Federal Research Center “Kazan Scientific Center of the Russian Academy of Sciences” (protocol No. 2 from 9 June 2022). Experiments were carried out on Wistar rats of both sexes purchased from the Laboratory Animal Breeding Facility (Branch of Shemyakin-Ovchinnikov Institute of Bioorganic Chemistry, Puschino, Moscow Region, Russia). Animals were kept in sawdust-lined plastic cages in a well-ventilated room at 20–22 °C in a 12 h light/dark cycle and 60–70% relative humidity and given ad libitum access to food and water.

Free rhodamine B or CFL16 1:1 PC cerasomes labeled with rhodamine B were intravenously injected in Wistar rats at the same dose (1.67 mg/kg of rhodamine B equivalent). Nontreated animals were used as control with respect to background fluorescence. Two hours after injection, animals were deeply anesthetized with isoflurane and exsanguinated by transcardial perfusion with cold phosphate-buffered saline (0.1 M, pH 7.4). Then animals were decapitated, and the brain was isolated and stored at −80 °C. Frozen tissue was moved to a −20 °C freezer 24 h before sectioning. Tissues were cut at 10 µm sections using Tissue-Tek Cryo3 microtome (Sakura Finetek, Torrance, CA, USA). Images were observed using a confocal laser scanning microscope Leica TSC SP5 MP (Leica, Wetzlar, Germany) using Cyanine 3 filter, excitation wavelength 550 nm and emission at 570 nm. Microphotographs were taken using the Leica LAS AF program.

### 4.10. Statistical Analysis

Measurements were repeated at least three times; mean and standard deviation were used to display the data. Statistical analysis was performed using the Mann–Whitney test (*p* < 0.01) [48].

## Figures and Tables

**Table 1:** DLS characteristics of hybrid cerasomes prepared via thin film hydration with varied composition of lipids and the effect of modification with 14-6-14(Et) on the zeta potential at pH = 7.4.

Sample Composition	D_h_, nm	PdI	Zeta Potential, mV
100% CFL16 0% PC	333 ± 20	0.300	−20 ± 0.3
90% CFL16 10% PC	252 ± 16	0.263	+16.1 ± 0.7 *
80% CFL16 20% PC	216 ± 8	0.275	−15.6 ± 0.2
70% CFL16 30% PC	203 ± 2	0.259	−14.6 ± 0.4
60% CFL16 40% PC	191 ± 8	0.265	−13.1 ± 0.2
50% CFL16 50% PC	153 ± 1	0.262	−12.2 ± 0.1
25% CFL16 75% PC	88 ± 3	0.245	−7.6 ± 0.4
0% CFL16 100% PC	89 ± 8	0.268	−7.0 ± 0.5
50% CFL16 50% PC + 1/35 14-6-14(Et)	161 ± 8	0.268	40.7 ± 0.8

*—measured at acidic pH.

**Table 2:** Hydrodynamic diameters and correlogram intercepts evaluated by DLS for hybrid PC-CFL16 cerasomes initially after 1 h and after 24 h of incubation with 5× molar excess of Triton X-100 surfactant.

Sample Composition	D_h_, nm			Intercept		
	Initial	1 h	24 h	Initial	1 h	24 h
80% CFL16 20% PC	216	188	258	0.741	0.743	0.519
70% CFL16 30% PC	203	184	222	0.750	0.763	0.370
60% CFL16 40% PC	191	144	184	0.706	0.778	0.490
50% CFL16 50% PC	153	117	129	0.789	0.793	0.719
25% CFL16 75% PC	88	102	190	0.739	0.537	0.158
0% CFL16 100% PC	89	73	52,212	0.664	0.492	0.189

**Table 3:** Cytotoxicity of hybrid cerasomes with different compositions, free PTX and PTX formulated in cerasomes toward T98G glioma cell line evaluated by 24 h MTT test.

Sample Composition	IC_50_ (CFL), μM	IC_50_ (PTX), μM
CFL 1 mM PC 1 mM	354.8 ± 32.7	
CFL 1 mM PC 1 mM Tw80 0.2 mM	320.6 ± 29.0	
CFL 1 mM PC 1 mM +14-6-14(Et)	341.2 ± 9.6	
PTX		8.8 ± 0.98
CFL 1 mM PC 1 mM + PTX	2.9 ± 0.07 *	0.24 ± 0.1 **
CFL 1 mM PC 1 mM Tw80 0.2 mM + PTX	3.3 ± 0.37 *	0.26 ± 0.04 **
CFL 1 mM PC 1 mM +14-6-14(Et) + PTX	4.6 ± 0.20 *	0.4 ± 0.04 **

Data are presented as mean ± SD of three independent experiments. * Values indicate *p* < 0.01 compared to CFL 1 mM PC 1 mM. ** Values indicated *p* < 0.01 compared to PTX.

## Data Availability

Analyzed data are included in this manuscript. Raw data are available from the authors upon request.

## Supplementary Materials

The supporting information can be downloaded at: https://www.mdpi.com/article/10.3390/ijms24043632/s1, see ref [49,50].
