# Supplementary material for: The Formation of Morphologically Stable Lipid Nanocarriers for Glioma Therapy
Source: Int J Mol Sci. 2023 Feb 11;24(4):3632. doi: 10.3390/ijms24043632 (PMC9964330; doi:10.3390/ijms24043632)
Supplement: Supplementary file 1 [file ijms-24-03632-s001.zip › ijms-2161303-supplementary.pdf]

Supplementary Information

The formation of morphologically stable lipid nanocarriers for glioma therapy

Rais Pavlov, Elvira Romanova, Denis Kuznetsov, Anna Lyubina, Syumbelya Amerhanova, Alexandra Voloshina, Daina Buzyurova, Vasily Babaev, Irina Zueva, Konstantin Petrov, Svetlana Lukashenko, Gulnara Gaynanova and Lucia Zakharova*

Arbuzov Institute of Organic and Physical Chemistry, FRC Kazan Scientific Center, Russian Academy of Sciences, 420088 Kazan, Russia

Correspondence: luciaz@mail.ru

Section S1

Table S1. DLS characteristics of hybrid DPPC CFL16 (1:1) cerasomes prepared by injection method at different temperatures and pre-hydrolysis time.

Sample	T, °C	$T_{hydrolysis}$, h	D_h, nm	PdI	Zeta potential, mV
CFL16	25	24	212	0.128	-45
CFL16 DPPC (1:1)	25	2	362	0.445	-31
CFL16 DPPC (1:1)	25	24	593	0.437	-23
CFL16 DPPC (1:1)	42	24	237	0.227	-14

DLS analysis of injection cerasomes was done on Malvern Zetasizer NanoZS (Malvern, USA) at 173° scattering angle, using a HeNe 633 nm laser at 25 °C

The ESI MS measurements were performed using an AmazonX ion trap mass spectrometer (Bruker Daltonic GmbH, Germany) in positive mode in the mass range of 70–2000. The capillary voltage was −3500 V, nitrogen drying gas – 8 L·min^{-1}, desolvation temperature – 250 °C. An acetonitrile/water solution (70:30) was used as a mobile phase at a flow rate of 0.2 mL/min by binary pump (Agilent 1260 chromatograph, USA).

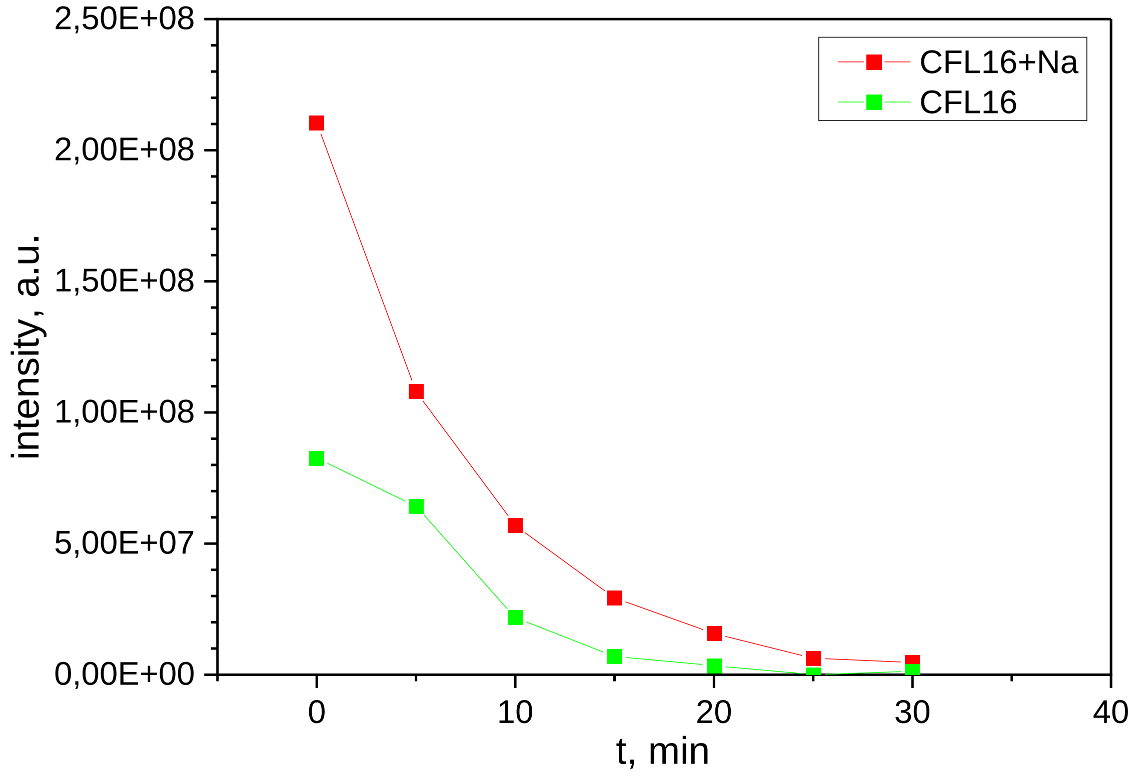

Figure S1. Non-hydrolyzed CFL16 concentration in EtOH with 1 mM HCl, 25 °C, over time.

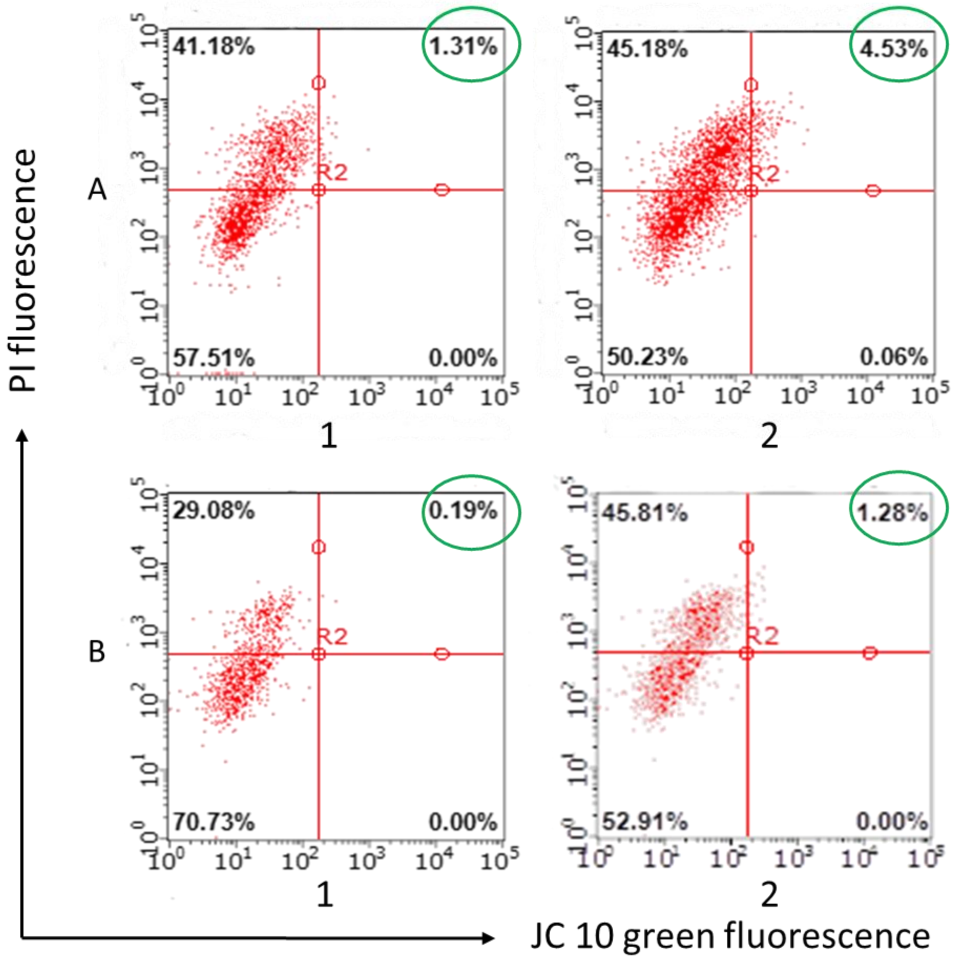

Figure S2. Apoptotic effects were measured by flow cytometry using annexin V-Alexa Fluor 647 staining protocol. Nanoparticle composition is as follows: (A) CFL 1 mM PC 1 mM; (B) CFL 1 mM PC 1 mM 14-6-14(Et) 0.057 mM; Samples were diluted and tested at 0.1 mM of CFL16 (column 1) and 0.2 mM of CFL16 (column 2) .

Hemolysis

Series of cerasome samples (or free PTX) were evaluated in terms of hemolytic activity by comparing the optical density of a sample treated erythrocyte suspension with the optical density of blood at 100% hemolysis [1]. 10% suspension of human erythrocytes was used as an object of investigation. An erythrocytic mass was treated with heparin and washed thrice with physiological saline (p.sal., 0.9% NaCl), centrifuged for 10 min at 800 rpm, and resuspended in p.sal to make a 10% suspension. 450 µL of the tested sample at the corresponding dilution was added to 50 µL erythrocyte suspension. The red blood cells were treated for 1 h at 37°C and then centrifuged for 10 min at 2000 rpm. Released hemoglobin was controlled by measuring the optical density of the supernatant on Microplate reader Invitrologic (Russia) at 540 nm. Control sample with 0% hemolysis was prepared by adding of 50 µL of 10% erythrocyte suspension to 450 µL of p. sal.,

and the 100% hemolysis control sample was prepared by adding of 50 µL of 10% red blood cell suspension to 450 µL of distilled water.

Table S2. Hemolysis caused by cerasomes of different composition and paclitaxel (PTX) itself*.

№	Sample	Concentration (PC) µM	Hemolysis, %	HC_{50} µM (PC)
1	CFL16 1mM PC 1mM	500	8.7	>500
		250	3.9	
		125	0	
2	CFL16 1mM PC 1 mM +14-6-14(Et)	500	7.2	>500
		250	2.7	
		125	0	
3	CFL16 1mM PC 1 mM +PTX	500	12.5	>500
		250	6.3	
		125	0	
4	CFL16 1mM PC 1 mM + 14-6-14(Et)+ PTX	500	7.7	>500
		250	2.8	
		125	1.6	

* – PTX was tested for hemolysis, and was found to induce 11.5% hemolysis at 100 µM, 5.8% at 50 µM and 0% at 25 µM.

Hemagglutination induction

In the experiment, human erythrocyte mass was used (Group IV). The erythrocytes were washed twice with 0.9% saline and centrifuged at 2500 g for 10 minutes at 4˚C. After each cycle, the supernatant was carefully removed. The erythrocytes were then resuspended in 0.9% saline to a concentration of 2%.

The hemagglutination activity of the test compounds was analyzed in a 96-well U microtiter plate. Next, two-fold serial dilutions of the studied compositions were prepared. 100 µL of each dilution was mixed with 100 µL of a 2% packed red cell solution (1:1) and added to a 96-well U-microtiter plate. Each dilution was analyzed in 2 parallel wells. Samples were kept for 1 hour at 37°C, and then hemagglutination was observed with the naked eye [2]. Sample photographs were taken using a Nikon Eclipse Ci-S microscope (Nikon, Tokyo, Japan).

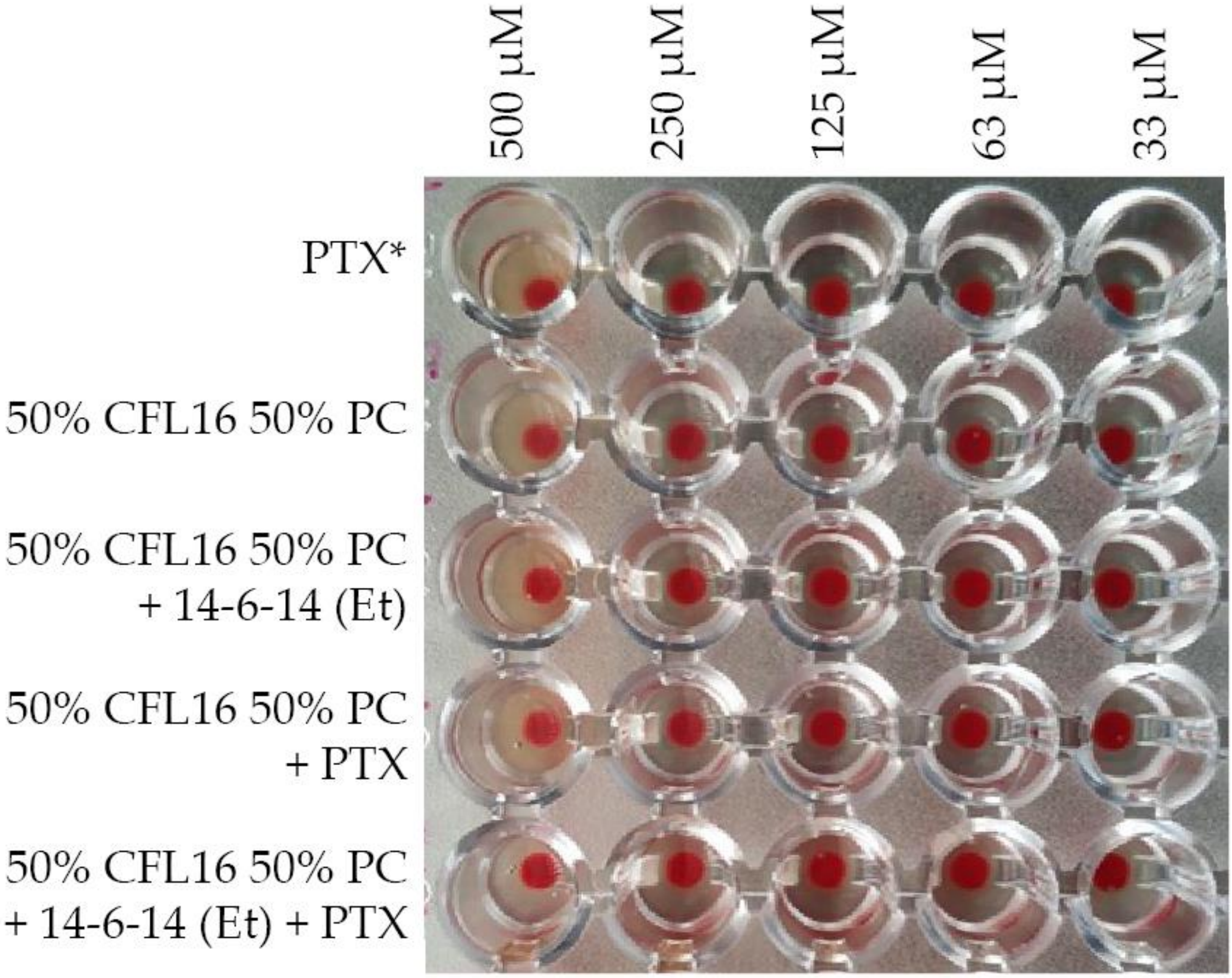

Figure S3. Induction of hemolysis caused by cerasomes of different compostion (cerasome-forming lipid (CFL16) and phosphatidylcholine (PC), each nanoparticle decorated with 10 mol% of Tween 80). Round red buttons of blood signify absence of agglutination. In case of agglutination a red carped of agglutinated blood covers the well [2]. Concentrations are given for the total amount of lipid forming the nanoparticles. * - Concentrations for paclitaxel (PTX) from left to right were as follows: 100 µM, 50 µM, 25 µM, 12.5 µM and 6.25 µM.

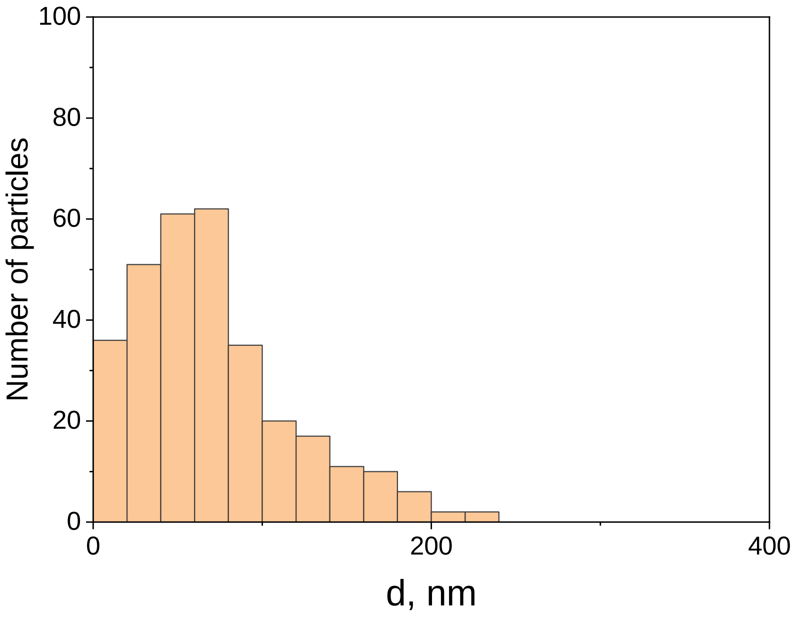

Figure S4. Particle diameter distribution in nm obtained from TEM image analysis.

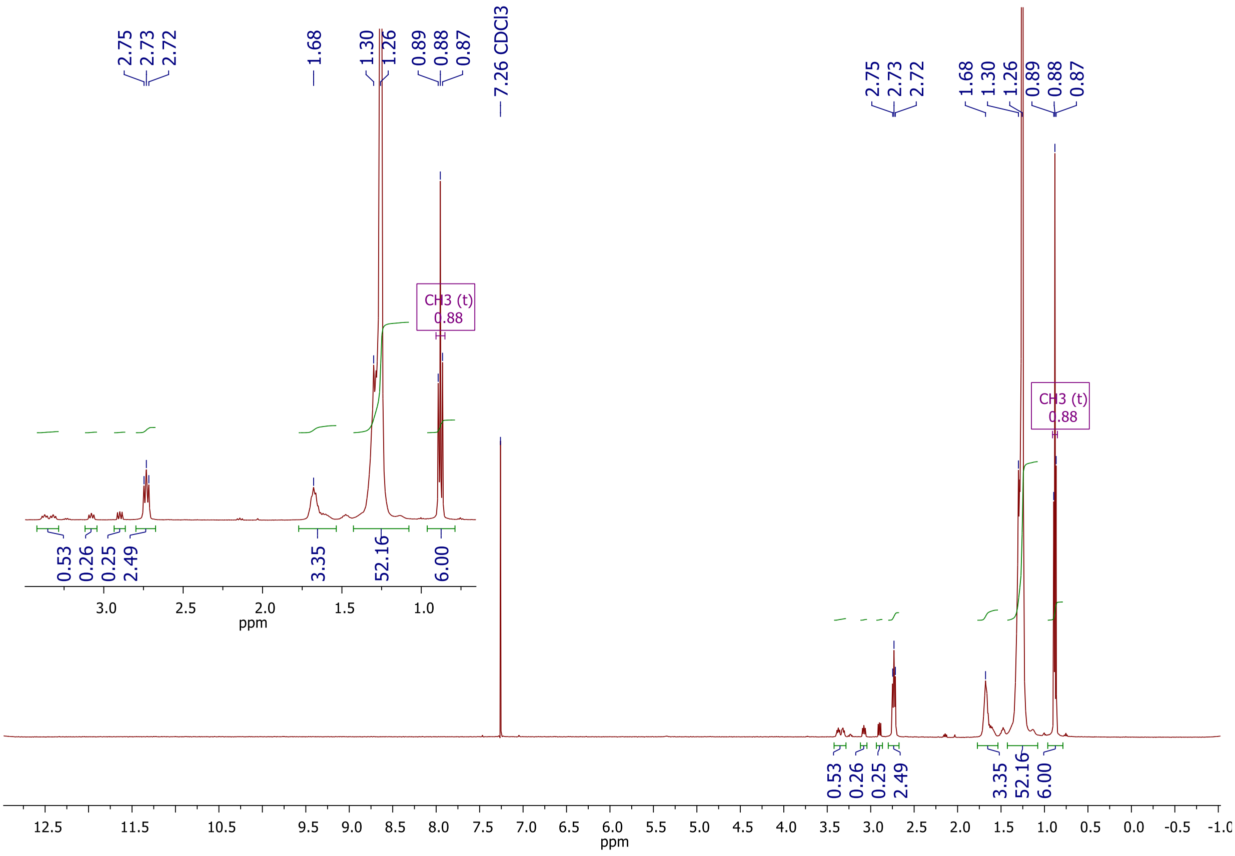

Figure S5. ^{1}H NMR spectrum of dihexadecylamine in $CDCl_3$; 30 °C.

^{1}H NMR spectrum (400 MHz, $CDCl_3$), δ, ppm (J, Hz): 0.88 t (CH_3, 6H, $^3J_{HH}$ 6.9); 1.30–1.26 m ($(CH_2)_{13}$, 52H); 1.68 m (N-CH_2-<u>CH_2</u>, 4H); 2.73 m (N-<u>CH_2</u>-CH_2, 4H).

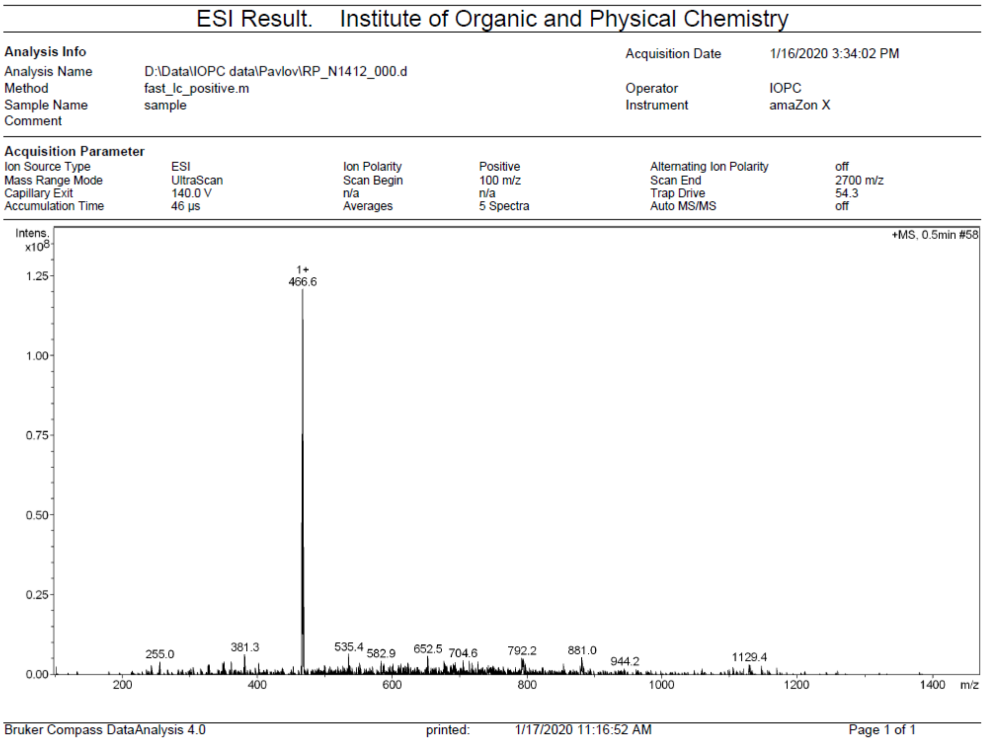

Figure S6. ESI mass spectrum of dihexadecylamine.

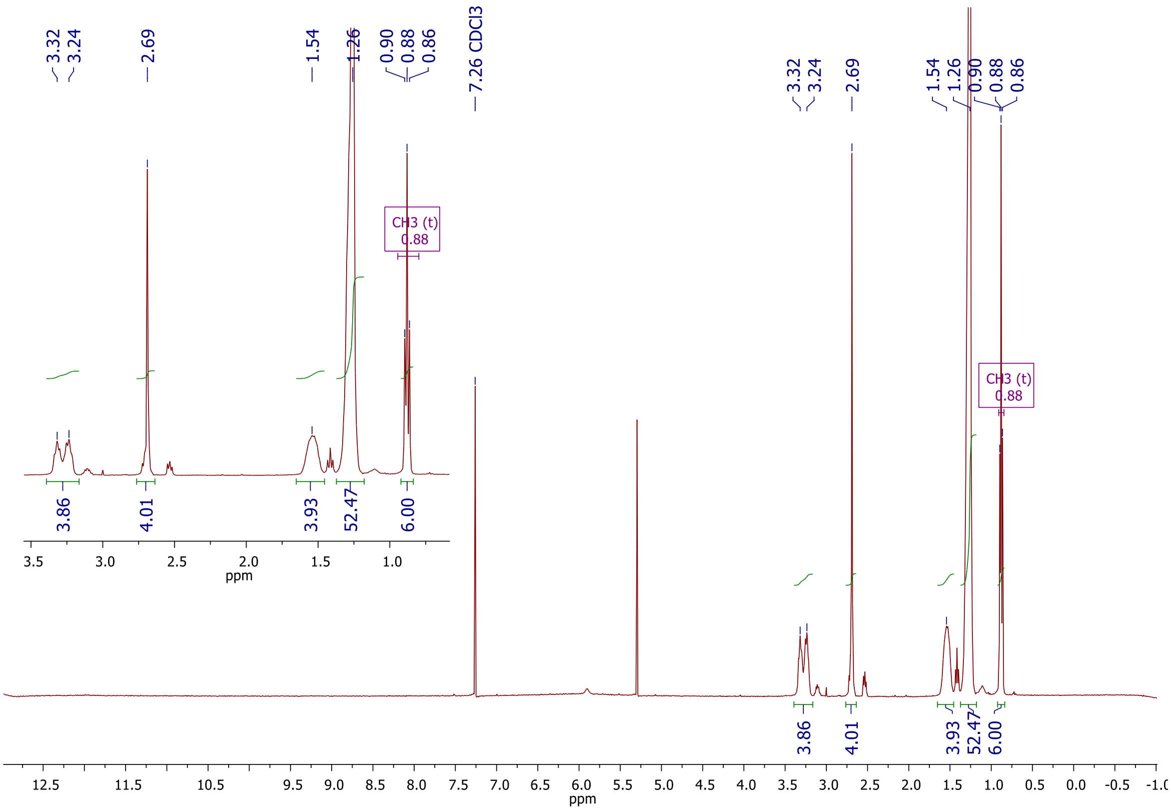

Figure S7. ^{1}H NMR spectrum of N,N-dihexadecylsuccinamic acid in $CDCl_3$; 30 °C.

^{1}H NMR spectrum (400 MHz, $CDCl_3$), δ, ppm (J, Hz): 0.88 t (CH_3, 6H, $^{3}J_{HH}$ 6.7); 1.26 m ($(CH_2)_{13}$, 52H); 1.54 m (N-CH_2-<u>CH_2</u>, 4H); 2.69 m (N-<u>CH_2</u>-CH_2, 4H); 3.24 m (N-C(O)-<u>CH_2</u>-CH_2-, 2H); 3.32 m (N-C(O)-CH_2-<u>CH_2</u>-, 2H).

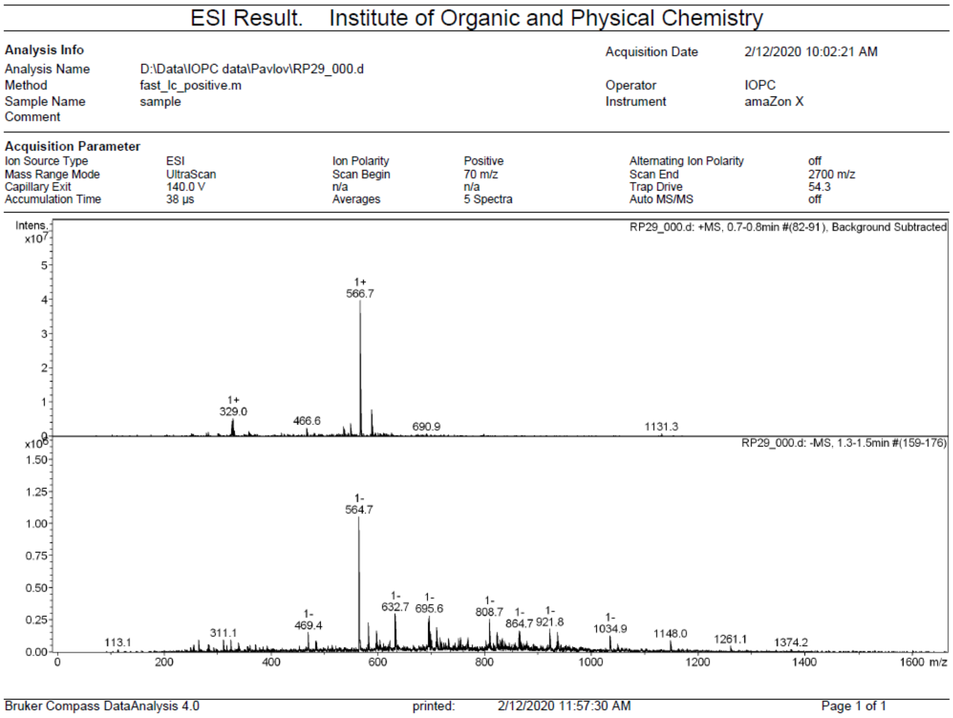

Figure S8. ESI mass spectrum of N,N-dihexadecylsuccinamic acid.

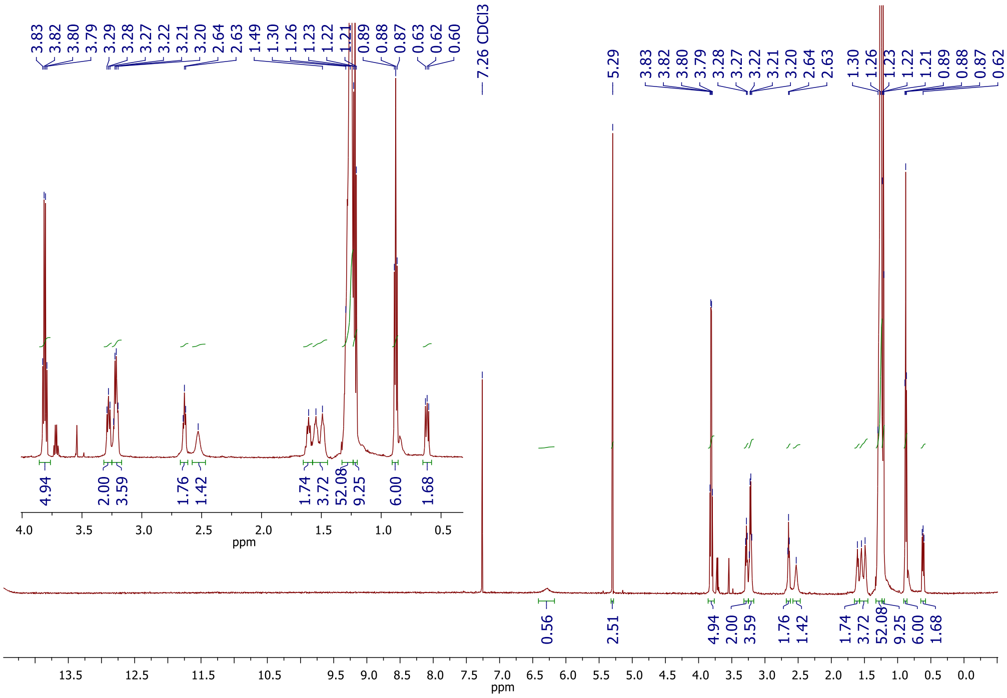

Figure S9. ^{1}H NMR spectrum of N-[N-(3-Triethoxysilyl)propylsuccinamoyl]dihexadecylamine (CFL16) in $CDCl_3$; 30 °C.

^{1}H NMR spectrum (400 MHz, $CDCl_3$), δ, ppm (J, Hz): 0.62 m (-NH-CH_2-CH_2-$\underline{CH}_2$-Si-, 2H); 0.88 t (CH_3, 6H, $^3J_{HH}$ 6.9); 1.22 t (-Si-O-CH_2-$\underline{CH}_3$, 9H, $^3J_{HH}$ 7.0); 1.30–1.26 m ($(CH_2)_{13}$, 52H); 1.55–1.49 two m (N-CH_2-$\underline{CH}_2$, 4H); 1.61 m (-NH-CH_2-$\underline{CH}_2$-CH_2-Si-, 2H); 2.53 m (-N-C(O)-$\underline{CH}_2$-CH_2-C(O)-NH-, 2H); 2.64 m (-N-C(O)-CH_2-$\underline{CH}_2$-C(O)-NH-, 2H); 3.22 m (N-$\underline{CH}_2$-CH_2, 4H); 3.28 m (-NH-$\underline{CH}_2$-CH_2-CH_2-Si-, 2H); 3.81 q (-Si-O-$\underline{CH}_2$-CH_3, 6H, $^3J_{HH}$ 7.0).

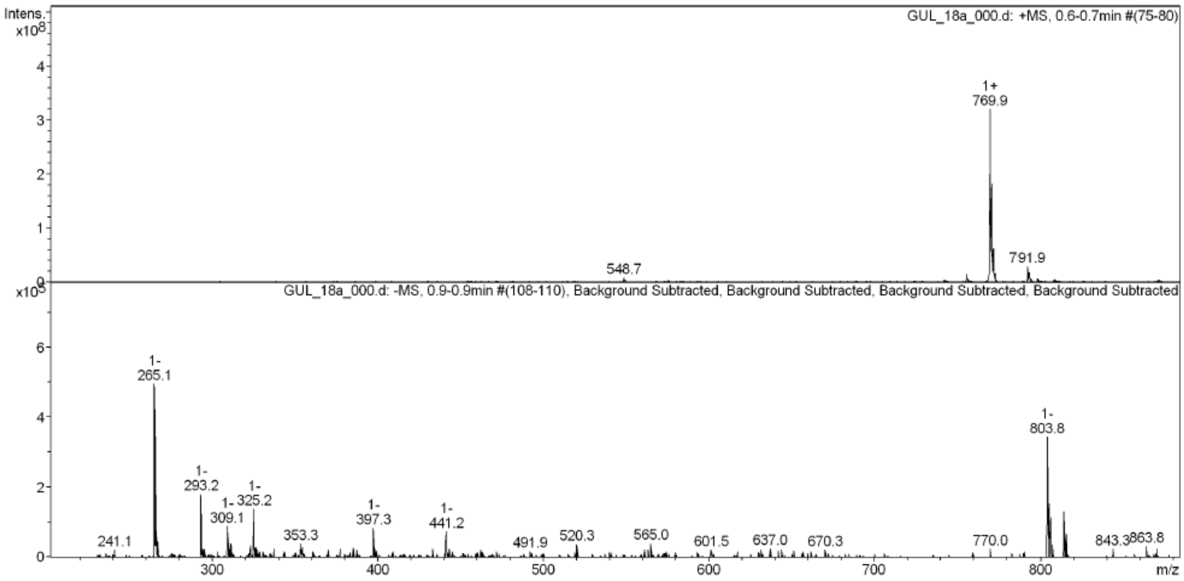

Figure S10. ESI mass spectrum of N-[N-(3-Triethoxysilyl)propylsuccinamoyl]dihexadecylamine (CFL16).

References

1. Sharipova, R.R.; Belenok, M.G.; Garifullin, B.F.; Sapunova, A.S.; Voloshina, A.D.; Andreeva, O.V.; Strobykina, I.Y.; Skvortsova, P.V.; Zuev, Y.F.; Kataev, V.E. Synthesis and Anti-Cancer Activities of Glycosides and Glycoconjugates of Diterpenoid Isosteviol. *MedChemComm* **2019**, *10*, 1488–1498, doi:10.1039/c9md00242a.
2. Banerjee, N.; Sengupta, S.; Roy, A.; Ghosh, P.; Das, K.; Das, S. Functional Alteration of a Dimeric Insecticidal Lectin to a Monomeric Antifungal Protein Correlated to Its Oligomeric Status. *PLoS ONE* **2011**, *6*, doi:10.1371/journal.pone.0018593.

Section S2: DLS data

Table 1 in manuscript. DLS characteristics of hybrid cerasomes prepared via thin film hydration with varied composition of lipids and the effect of modification with 14-6-14(Et) on the zeta potential at pH = 7.4.

Number	Sample composition	D_h, nm	PdI	Zeta potential, mV
1	100% CFL16 0% PC	333 ± 20	0.300	-20 ± 0.3
2	90% CFL16 10% PC	252 ± 16	0.263	+16.1 ± 0.7
3	80% CFL16 20% PC	216 ± 8	0.275	-15.6 ± 0.2
4	70% CFL16 30% PC	203 ± 2	0.259	-14.6 ± 0.4
5	60% CFL16 40% PC	191 ± 8	0.265	-13.1 ± 0.2
6	50% CFL16 50% PC	153 ± 1	0.262	-12.2 ± 0.1
7	25% CFL16 75% PC	88 ± 3	0.245	-7.6 ± 0.4

8	0% CFL16 100% PC	89 ± 8	0.268	-7.0 ± 0.5
9	50% CFL16 50% PC + 1/35 14-6-14(Et)	161 ± 8	0.268	40.7 ± 0.8

Each correlogram corresponds to a sample number in the table and represents 3 separate measurements (red, green, yellow lines)

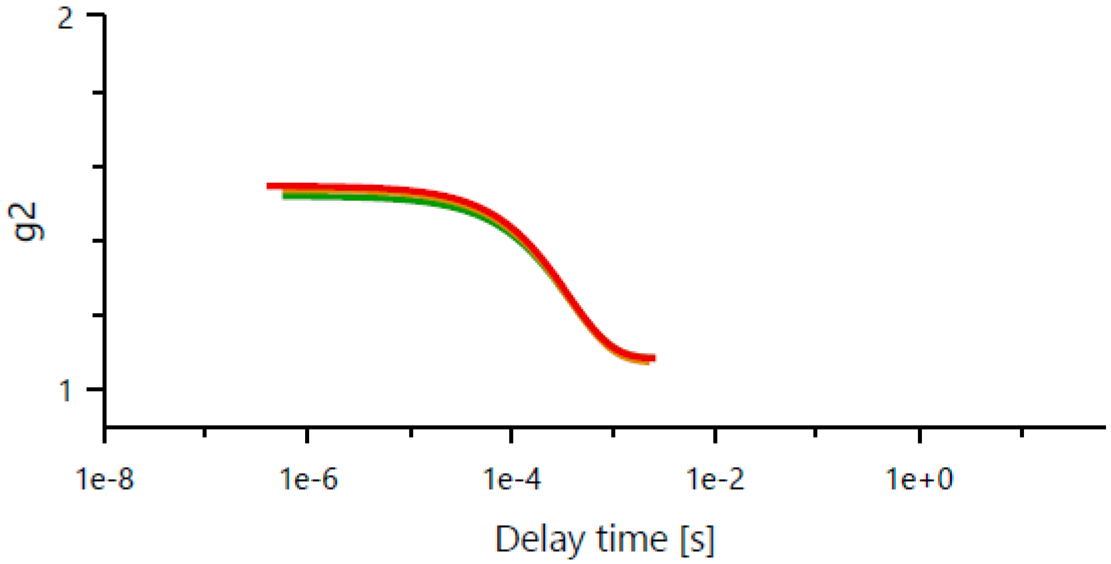

Sample 1

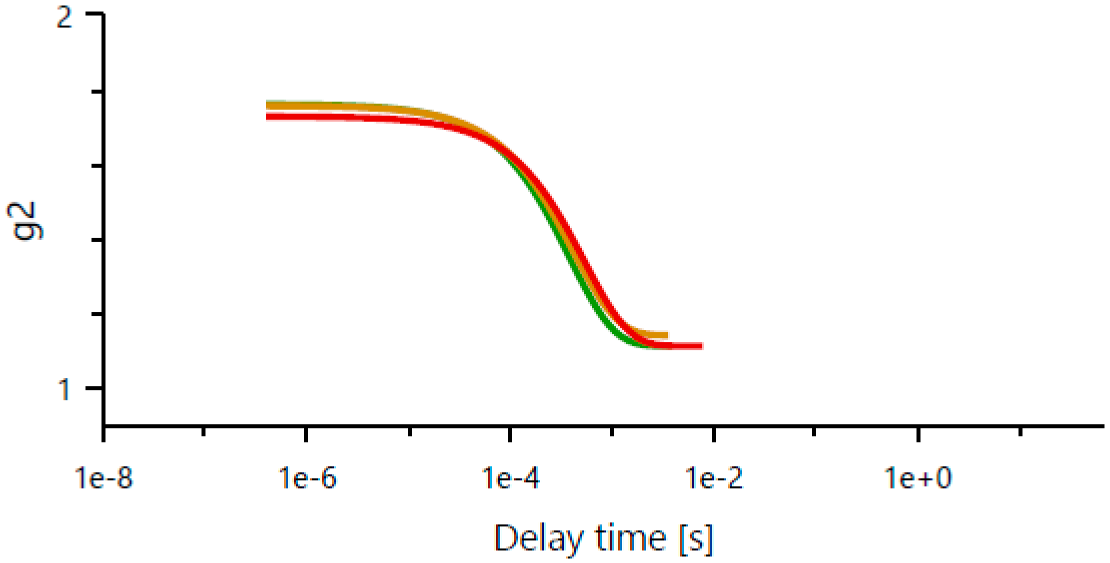

Sample 2

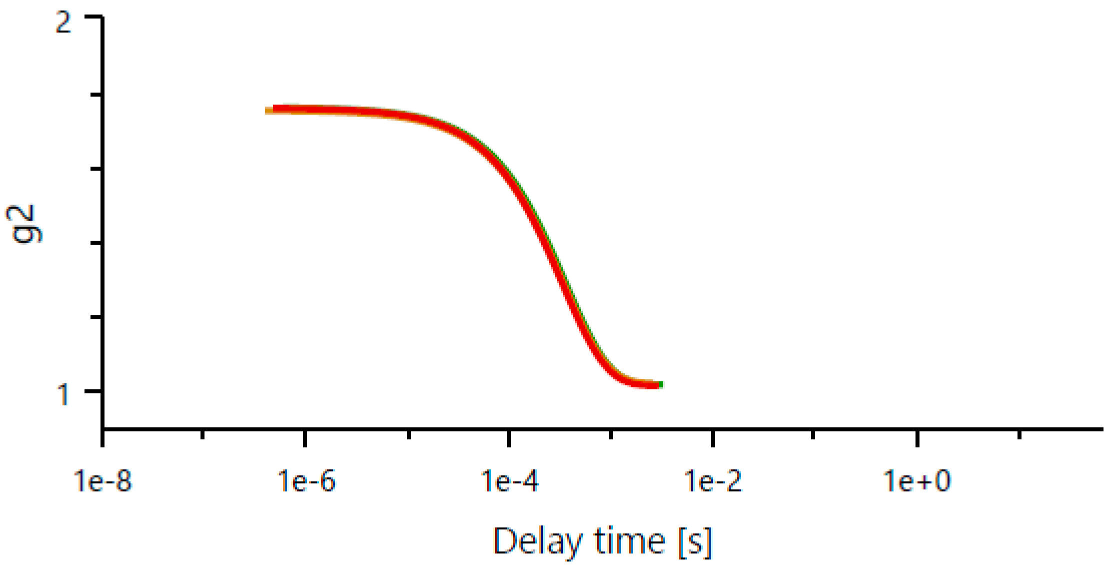

Sample 3

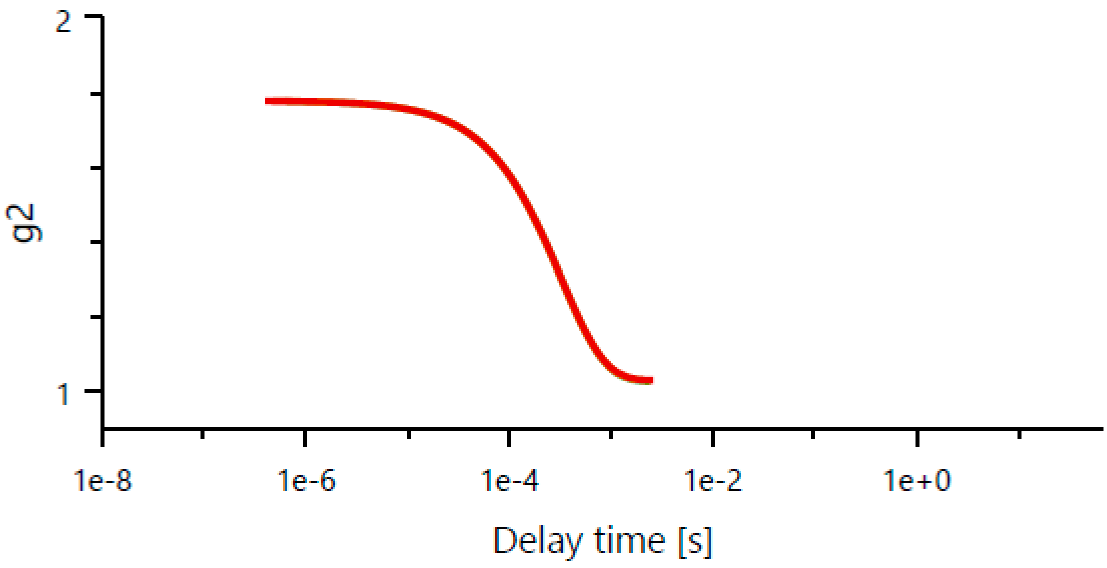

Sample 4

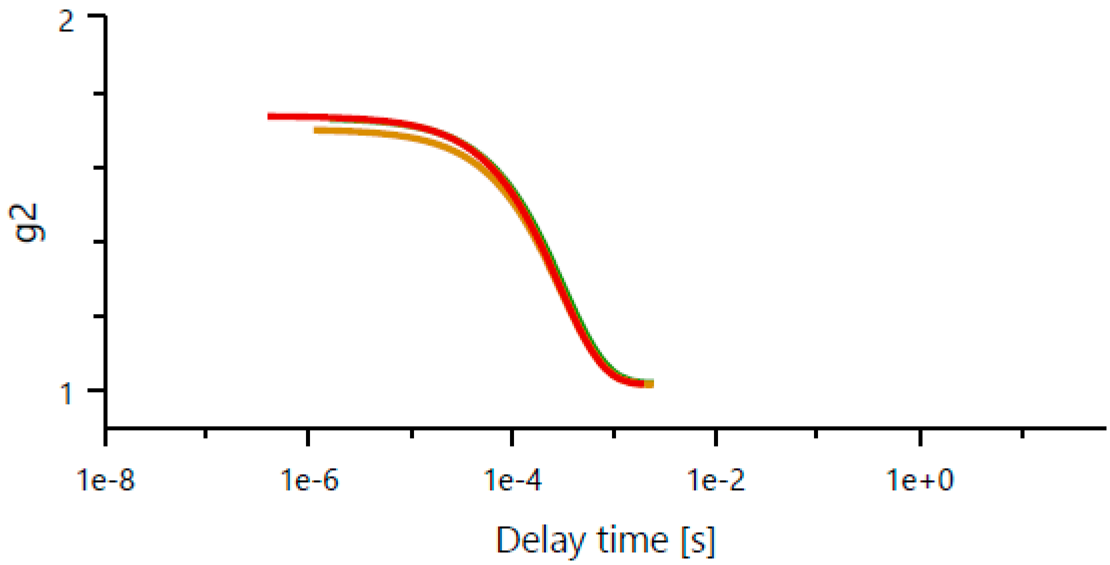

Sample 5

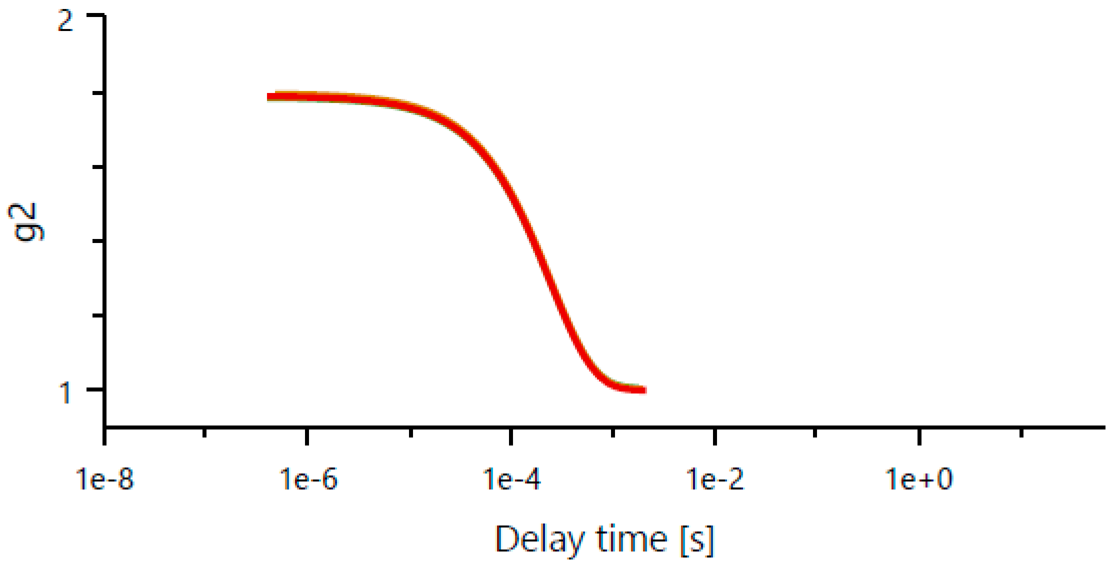

Sample 6

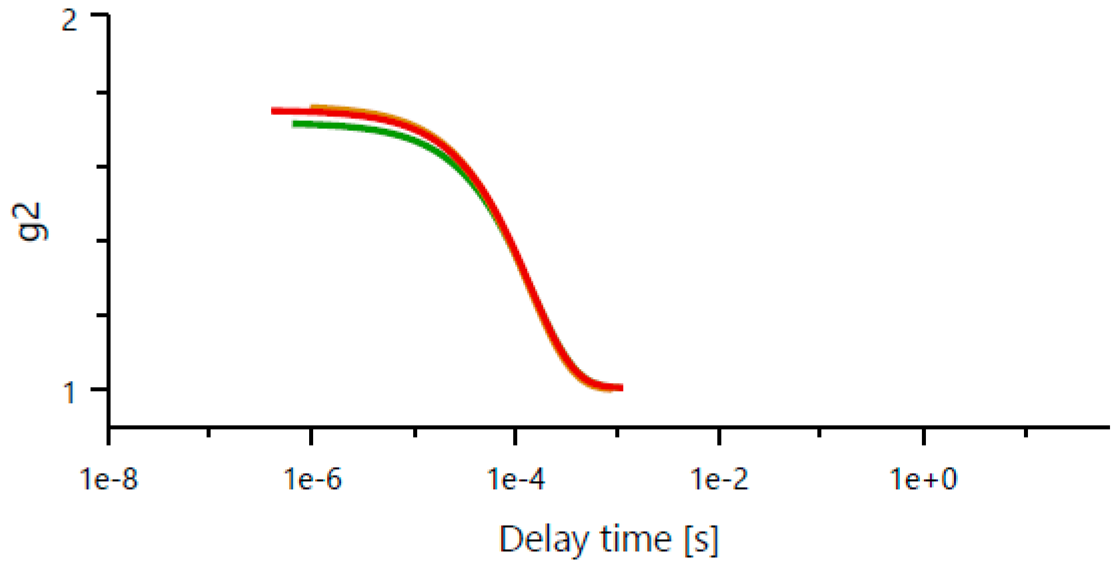

Sample 7

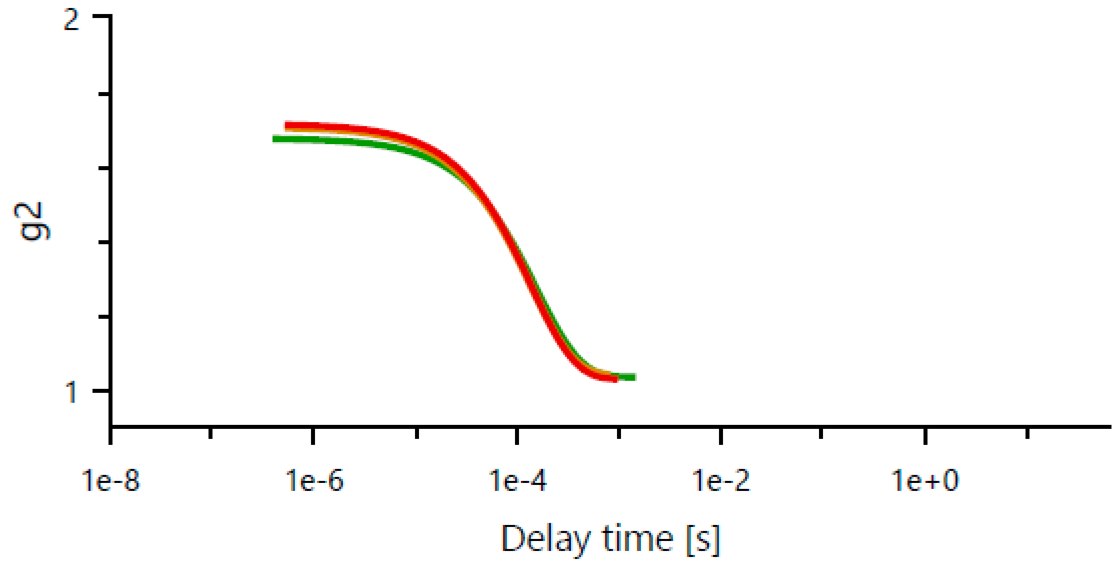

Sample 8

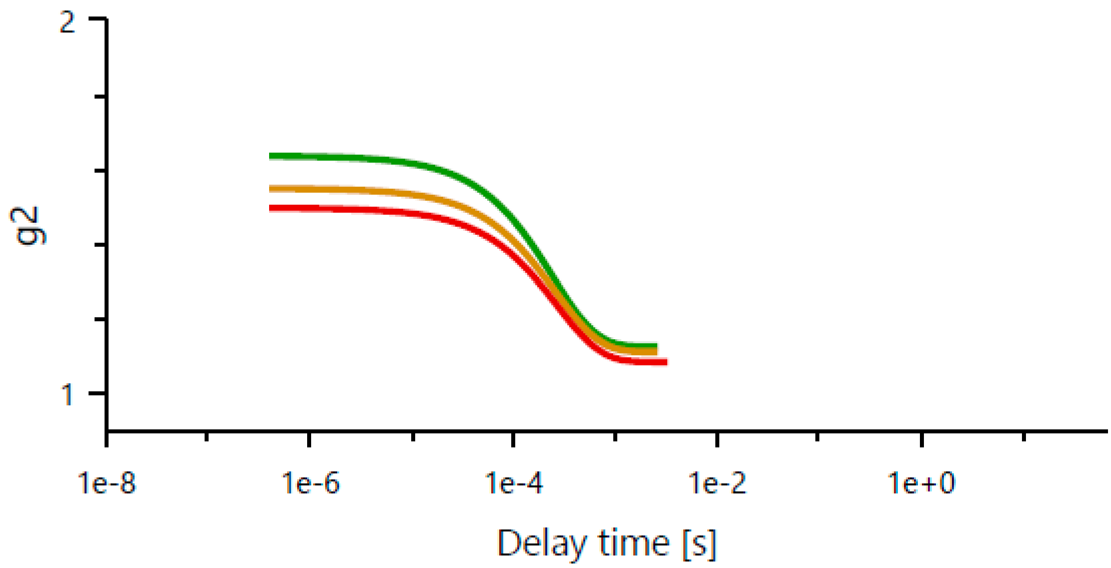

Sample 9

Table S1 in Supplementary Information. DLS characteristics of hybrid DPPC-CFL16 (1:1) cerasomes prepared by injection method at different temperatures and pre-hydrolysis time.

№	Sample	T, °C	$T_{hydrolysis}$, h	D_h, nm	PdI	Zeta potential, mV
1	CFL16	25	24	212	0.128	-45
2	CFL16 DPPC (1:1)	25	2	362	0.445	-31
3	CFL16 DPPC (1:1)	25	24	593	0.437	-23
4	CFL16 DPPC (1:1)	42	24	237	0.227	-14

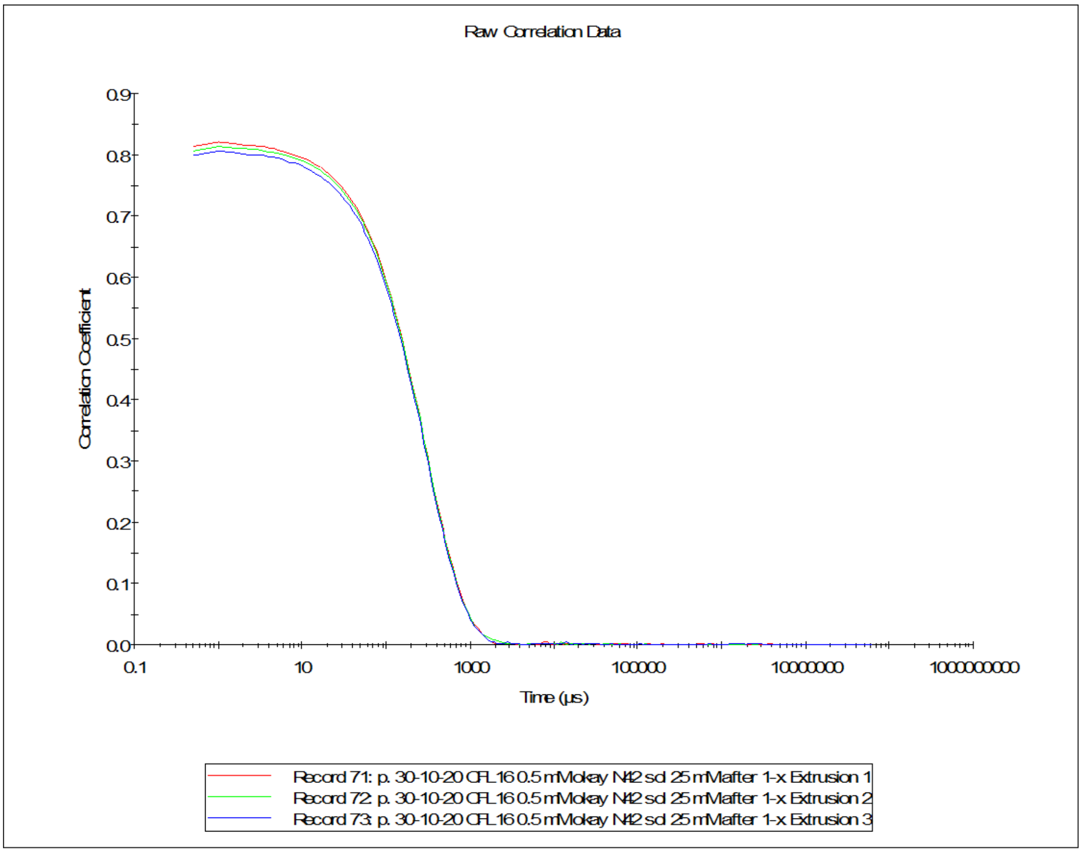

Sample 1

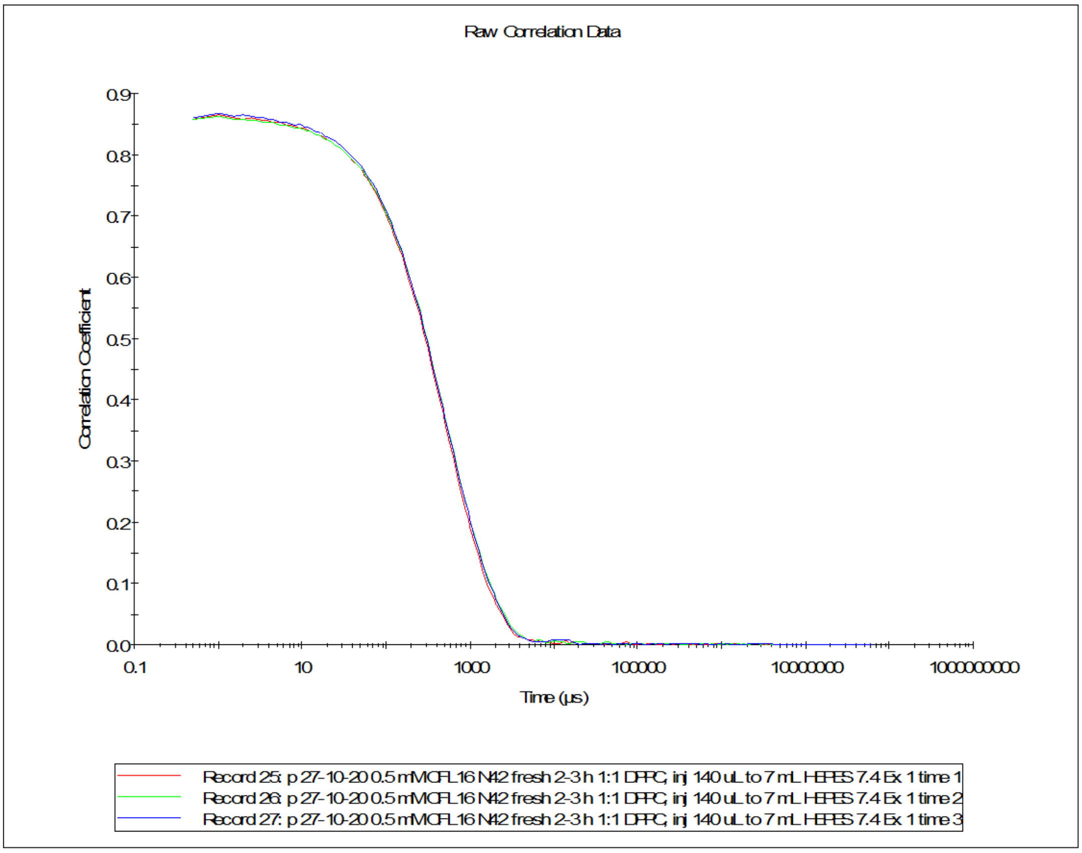

Sample 2

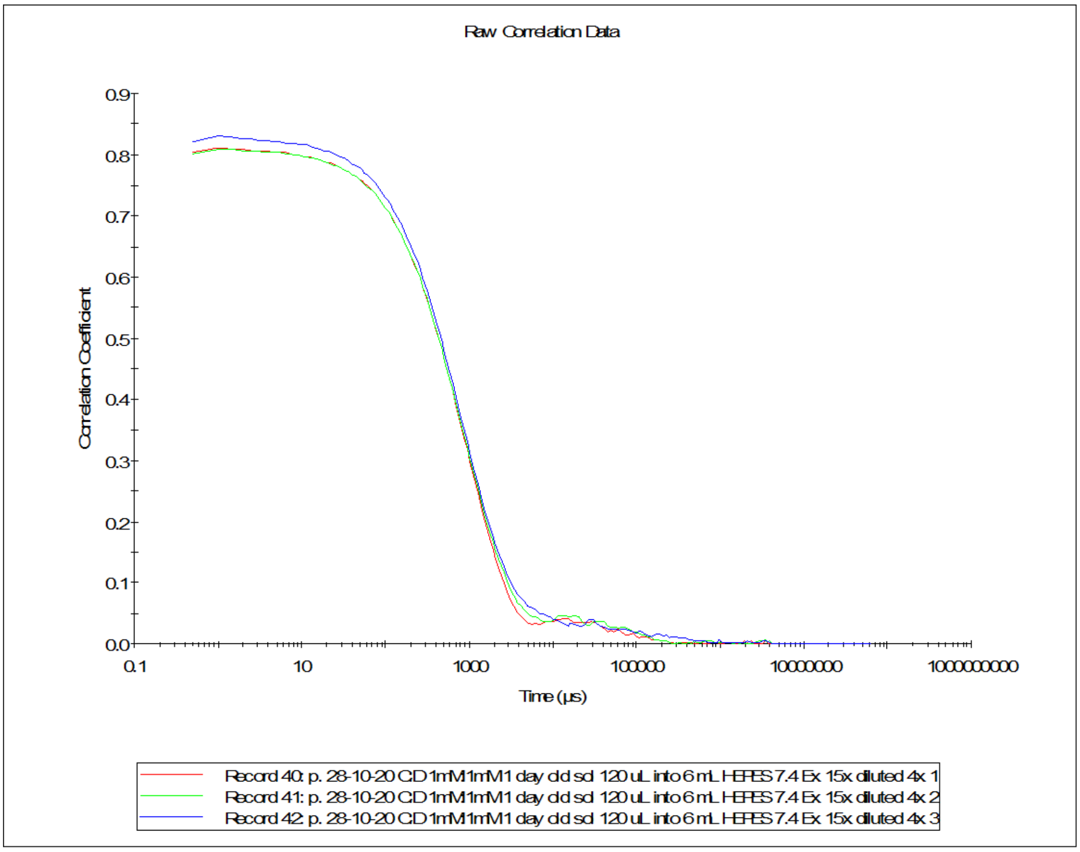

Sample 3

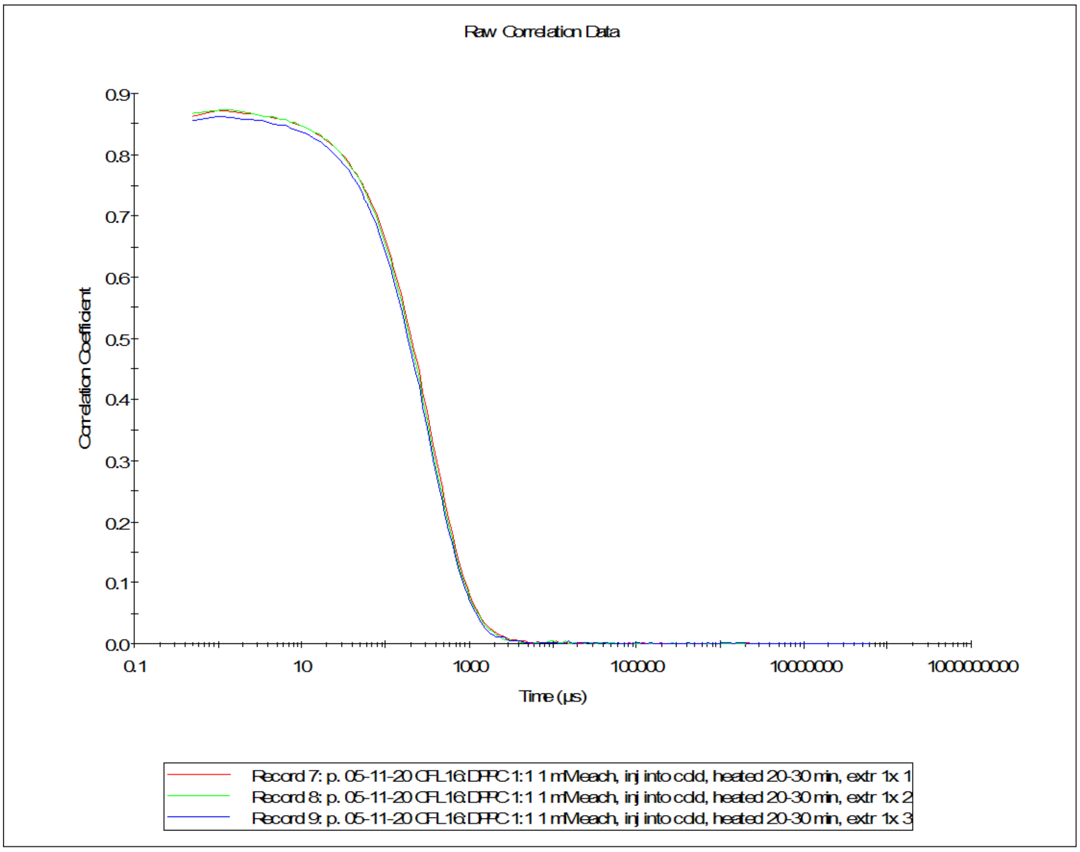

Sample 4

Section 3: Fluorescence microscopy

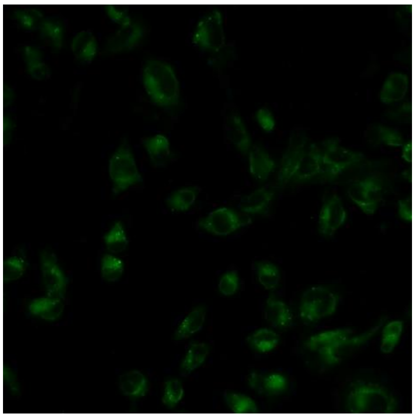	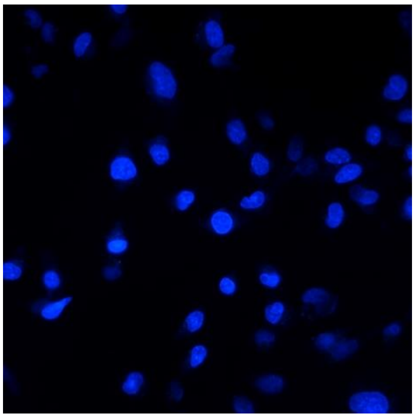	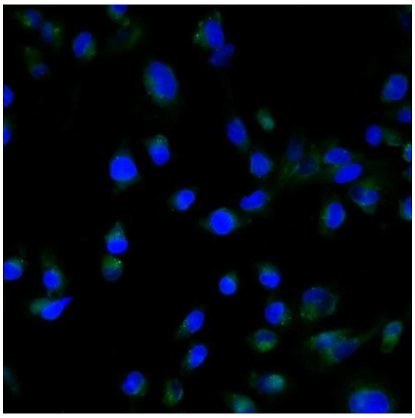
Control 2 h green	Control 2 h blue	Control 2 h merge
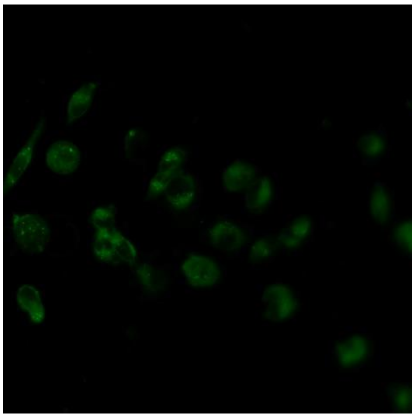	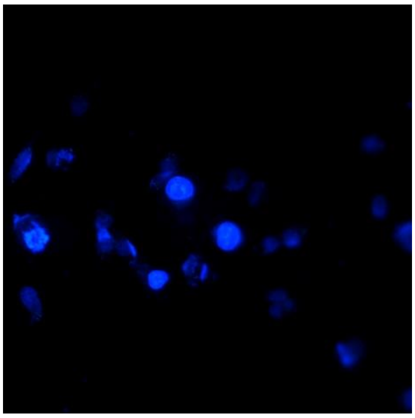	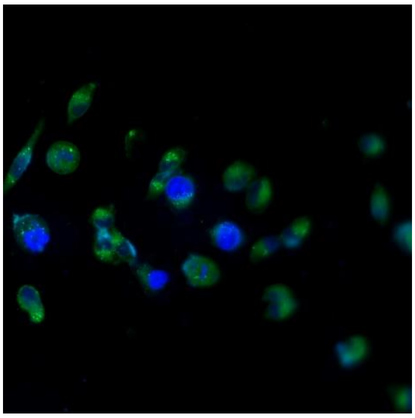
Control 6 h green	Control 6 h blue	Control 6 h merge
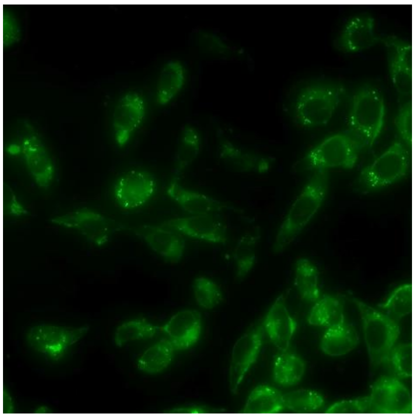	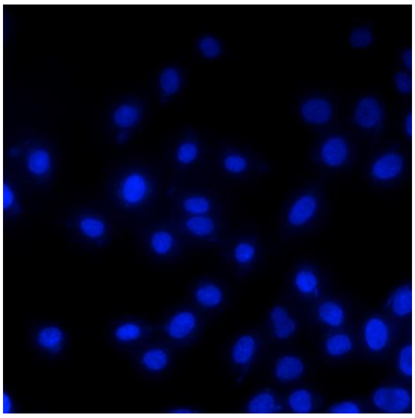	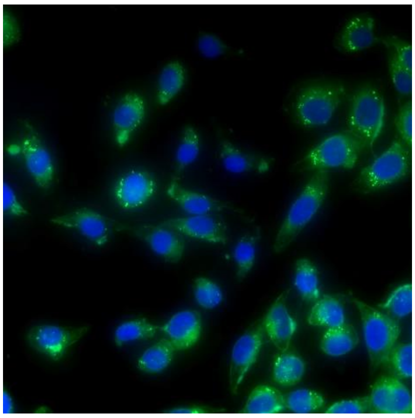
Control 24 h green	Control 24 h blue	Control 24 h merge

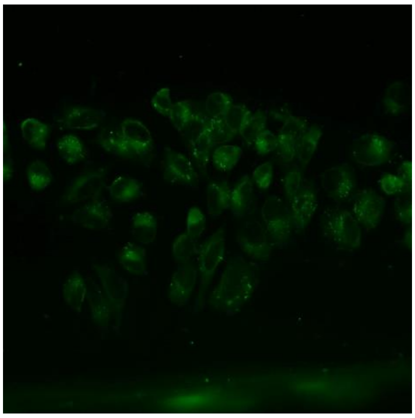	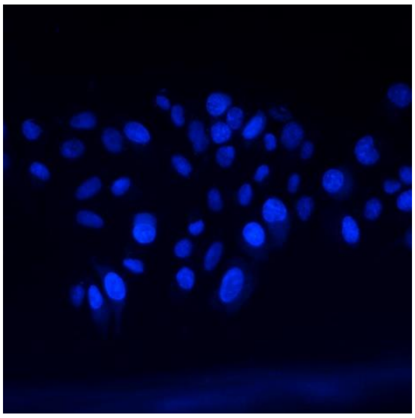	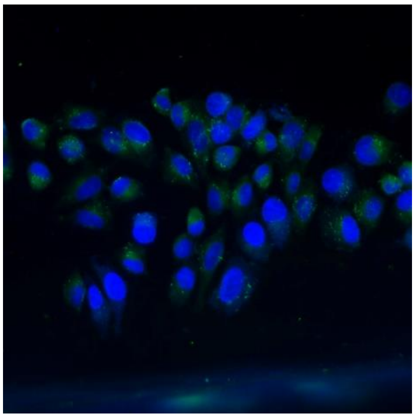
CFL16 PC 2 h green	CFL16 PC 2 h blue	CFL16 PC 2 h merge
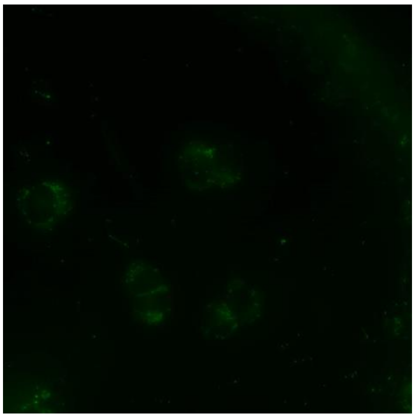	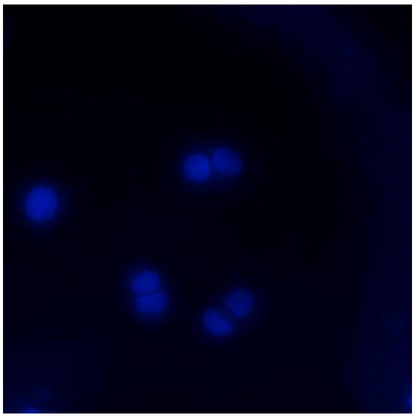	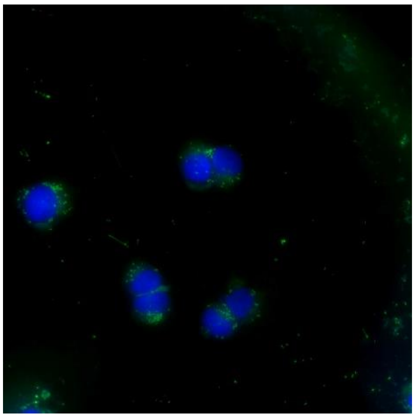
CFL16 PC 6 h green	CFL16 PC 6 h blue	CFL16 PC 6 h merge
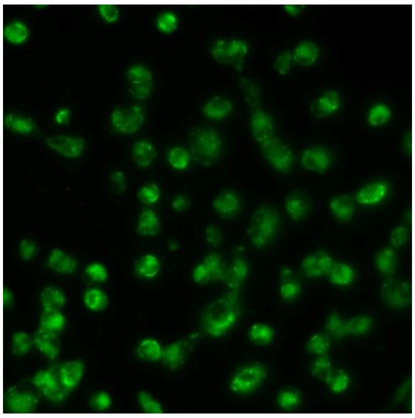	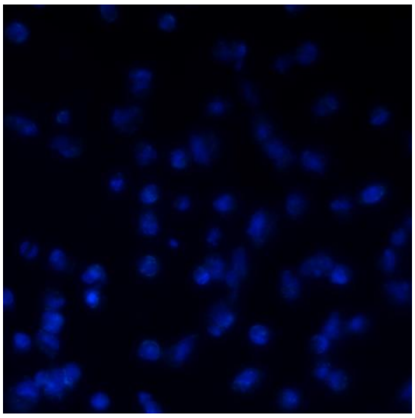	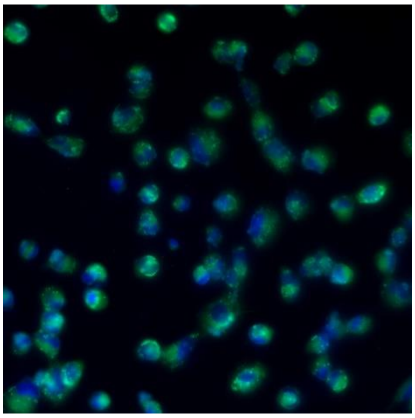
CFL16 PC 24 h green	CFL16 PC 24 h blue	CFL16 PC 24 h merge

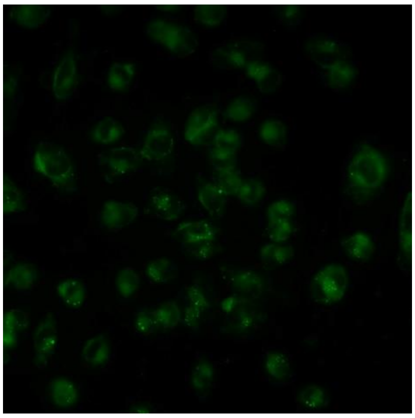	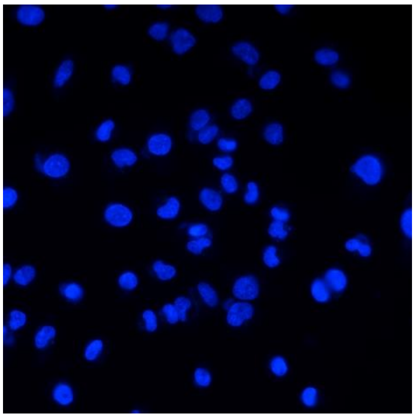	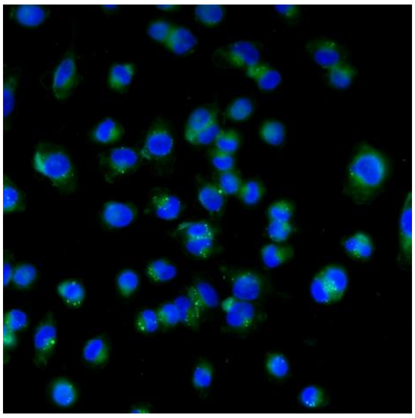
CFL16 PC 14-6-14(Et)<br>2 h green	CFL16 PC 14-6-14(Et)<br>2 h blue	CFL16 PC 14-6-14(Et)<br>2 h merge
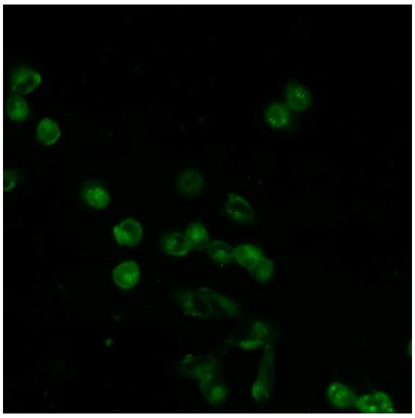	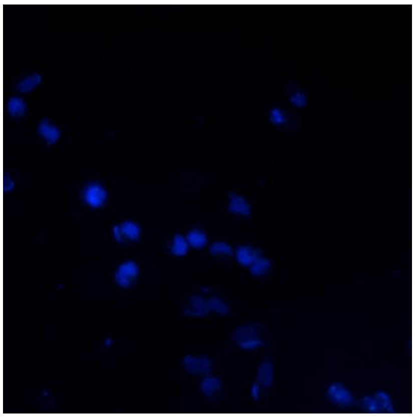	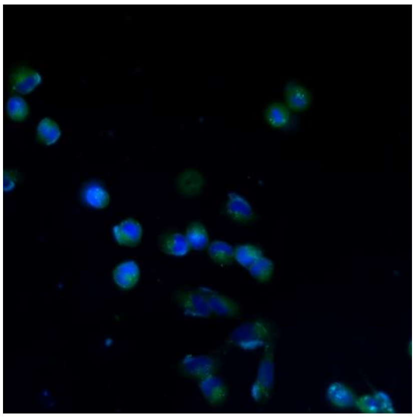
CFL16 PC 14-6-14(Et)<br>6 h green	CFL16 PC 14-6-14(Et)<br>6 h blue	CFL16 PC 14-6-14(Et)<br>6 h merge
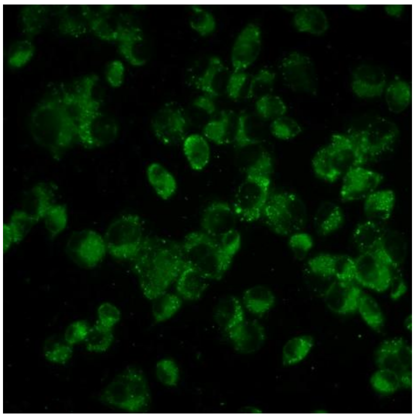	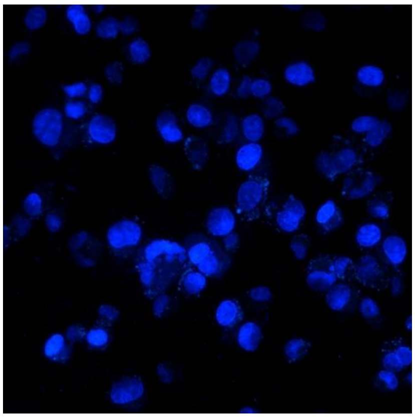	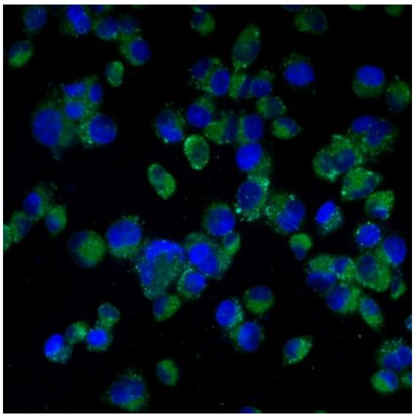
CFL16 PC 14-6-14(Et)<br>24 h green	CFL16 PC 14-6-14(Et)<br>24 h blue	CFL16 PC 14-6-14(Et)<br>24 h merge

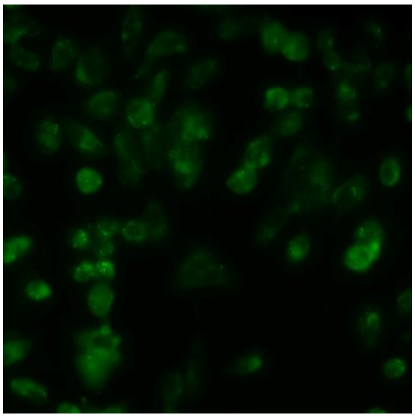	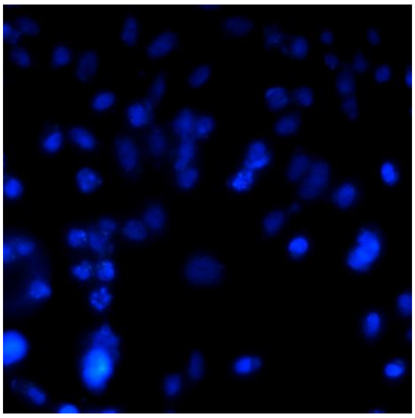	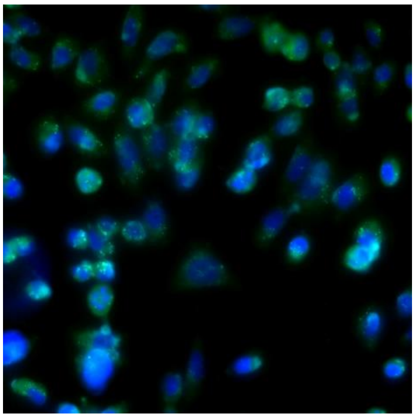
PTX 2 h green	PTX 2 h blue	PTX 2 h merge
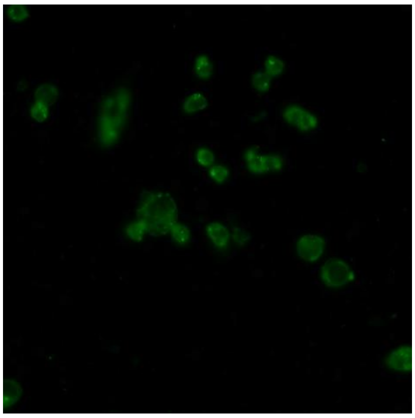	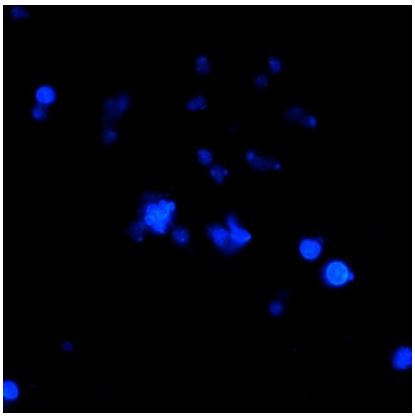	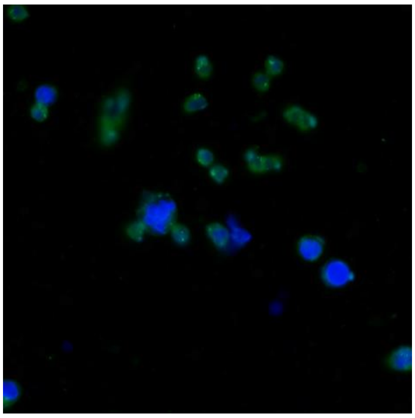
PTX 6 h green	PTX 6 h blue	PTX 6 h merge
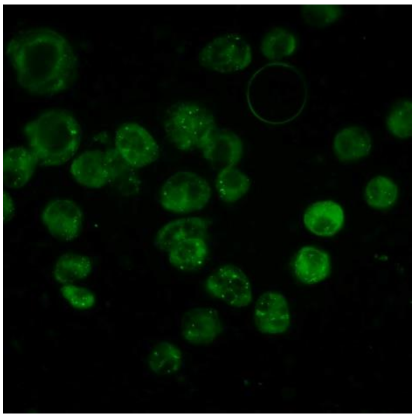	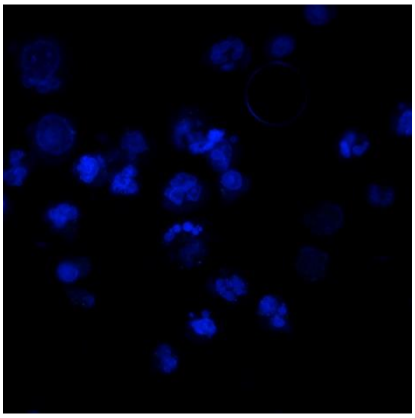	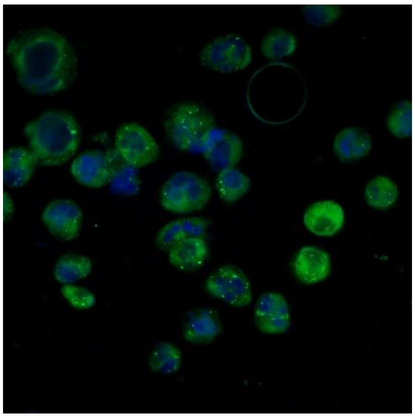
PTX 24 h green	PTX 24 h blue	PTX 24 h merge

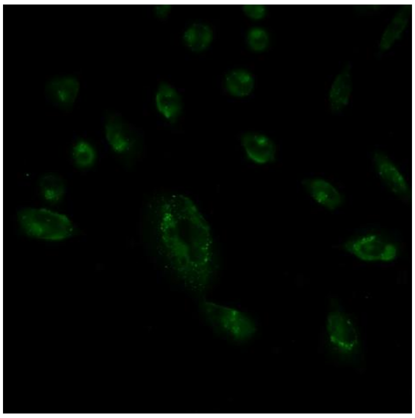	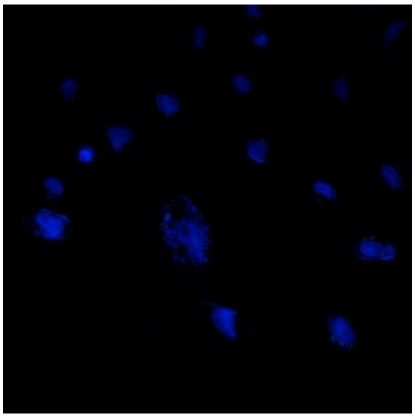	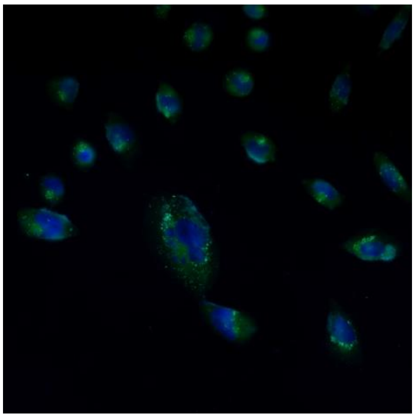
CFL16 PC PTX 2 h green	CFL16 PC PTX 2 h blue	CFL16 PC PTX 2 h merge
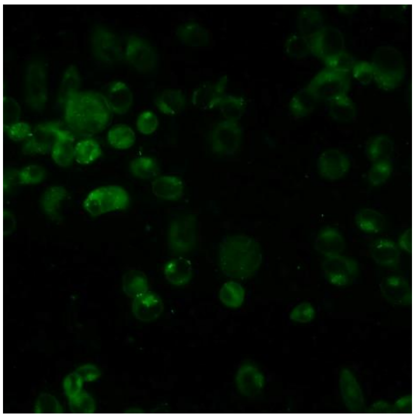	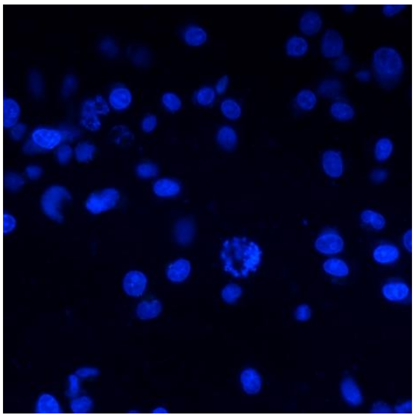	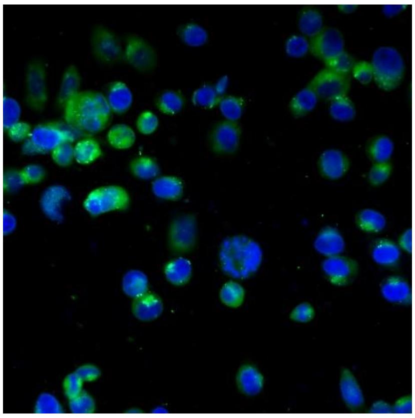
CFL16 PC PTX 6 h green	CFL16 PC PTX 6 h blue	CFL16 PC PTX 6 h merge
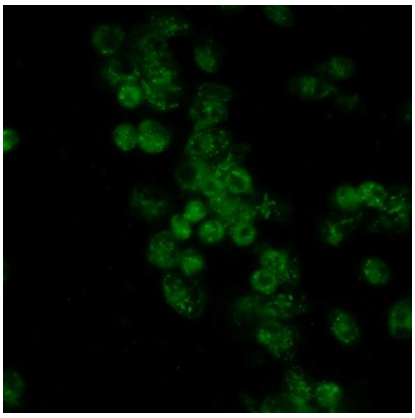	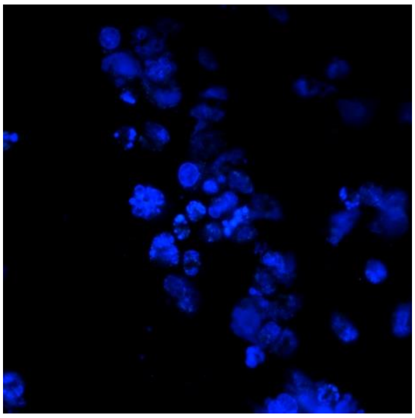	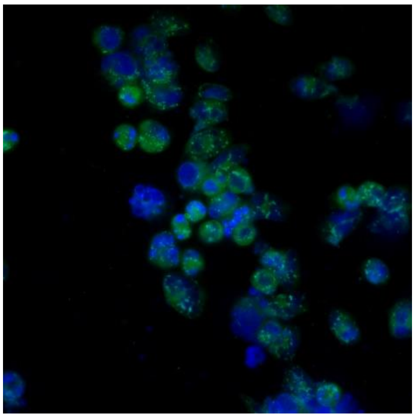
CFL16 PC PTX 24 h green	CFL16 PC PTX 24 h blue	CFL16 PC PTX 24 h merge

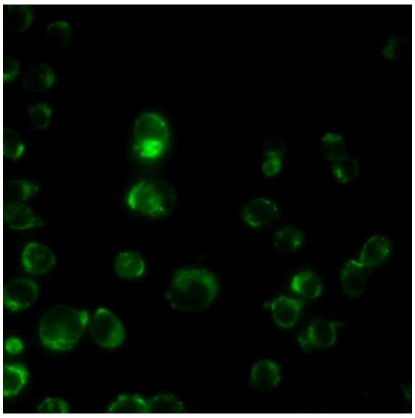	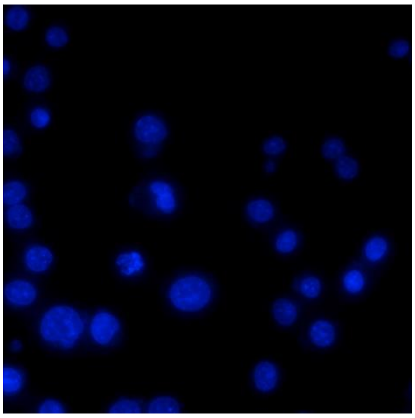	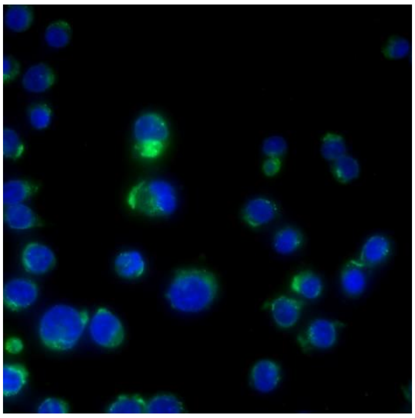
CFL16 PC 14-6-14(Et) PTX 2 h green	CFL16 PC 14-6-14(Et) PTX 2 h blue	CFL16 PC 14-6-14(Et) PTX 2 h merge
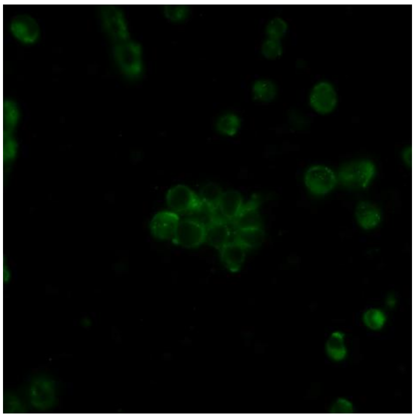	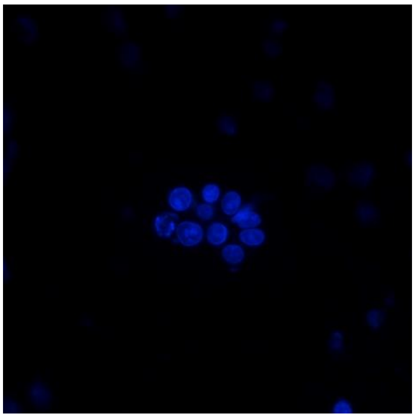	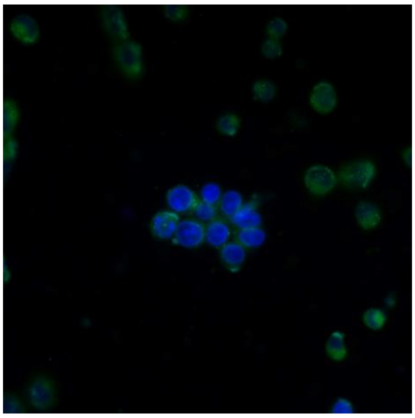
CFL16 PC 14-6-14(Et) PTX 6 h green	CFL16 PC 14-6-14(Et) PTX 6 h blue	CFL16 PC 14-6-14(Et) PTX 6 h merge
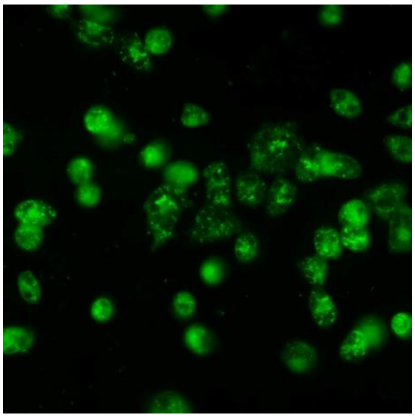	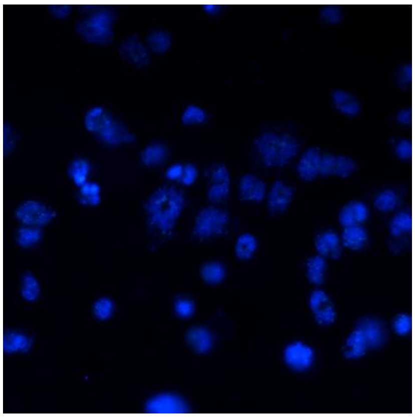	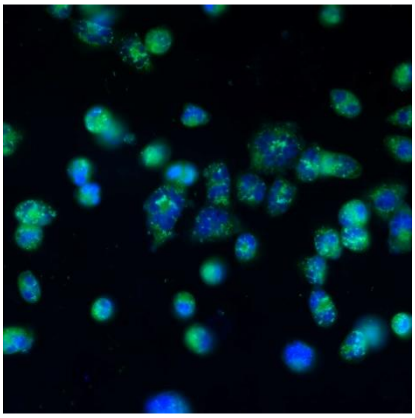
CFL16 PC 14-6-14(Et) PTX 24 h green	CFL16 PC 14-6-14(Et) PTX 24 h blue	CFL16 PC 14-6-14(Et) PTX 24 h merge
